# Evaluation of Antiviral, Antibacterial and Antiproliferative Activities of the Endophytic Fungus *Curvularia papendorfii*, and Isolation of a New Polyhydroxyacid †

**DOI:** 10.3390/microorganisms8091353

**Published:** 2020-09-04

**Authors:** Afra Khiralla, Rosella Spina, Mihayl Varbanov, Stéphanie Philippot, Pascal Lemiere, Sophie Slezack-Deschaumes, Philippe André, Ietidal Mohamed, Sakina Mohamed Yagi, Dominique Laurain-Mattar

**Affiliations:** 1 Université de Lorraine, CNRS, L2CM, F-54000 Nancy, France; aafraa21@hotmail.com (A.K.); mihayl.varbanov@univ-lorraine.fr (M.V.); stephanie.philippot@univ-lorraine.fr (S.P.); pascal.lemiere@univ-lorraine.fr (P.L.); 2 Botany Department, Faculty of Sciences and Technologies, Shendi University, P.O. Box 142 Shendi, Sudan; 3 Université de Lorraine, Inra, LAE, F-54000 Nancy, France; sophie.deschaumes@univ-lorraine.fr; 4 Université de Strasbourg, UMR 7021 CNRS, 67401 Illkirch, France; philippe.andre@unistra.fr; 5 Department of Botany, Faculty of Science, University of Khartoum, 11115 Khartoum, Sudan; ietidalem11@gmail.com (I.M.); sakinayagi@gmail.com (S.M.Y.)

**Keywords:** *Curvularia papendorfii*, endophytic fungi, human coronavirus HCoV 229E, *Staphylococcus* sp., MRSA, antiproliferative activity, polyhydroxyacid, kheiric acid

## Abstract

An endophytic fungus isolated from *Vernonia amygdalina,* a medicinal plant from Sudan, was taxonomically characterized as *Curvularia papendorfii.* Ethyl acetate crude extract of *C. papendorfii* revealed an important antiviral effect against two viral pathogens, the human coronavirus HCoV 229E and a norovirus surrogate, the feline coronavirus FCV F9. For the last one, 40% of the reduction of the virus-induced cytopathogenic effect at lower multiplicity of infection (MOI) 0.0001 was observed. Selective antibacterial activity was obtained against *Staphylococcus* sp. (312 µg/mL), and interesting antiproliferative activity with half maximal inhibitory concentration (IC_50_) value of 21.5 ± 5.9 µg/mL was observed against human breast carcinoma MCF7 cell line. Therefore, *C. papendorfii* crude extract was further investigated and fractionated. Twenty-two metabolites were identified by gas chromatography coupled to mass spectrometry (GC–MS), and two pure compounds, mannitol and a new polyhydroxyacid, called kheiric acid, were characterized. A combination of spectroscopic methods was used to elucidate the structure of the new aliphatic carboxylic acid: kheiric acid (3,7,11,15-tetrahydroxy-18-hydroxymethyl-14,16,20,22,24-pentamethyl-hexacosa-4E,8E,12E,16,18-pentaenoic acid). Kheiric acid showed an interesting result with a minimum inhibitory concentration (MIC) value of 62.5 µg/mL against meticillin-resistant *Staphylococcus aureus* (MRSA). Hence, endophytes associated with medicinal plants from Sudan merit more attention, as they could be a treasure of new bioactive compounds.

## 1. Introduction

Currently, the priority in research is the discovery of alternative treatments for viral and bacterial infections and cancer diseases. For human coronavirus (HCoV) and noroviruses, there is no vaccine or effective antivirals to prevent or control infections. Human coronaviruses (HCoVs) are a set of viruses that induce respiratory disease of varying severity, including common cold and pneumonia [1,2]. This wide-ranging family of viruses infects many species of mammals, including humans [3]. Their ability for interspecies transmission has led to the emergence of the Severe Acute Respiratory Syndrome (SARS) [4] and the Middle East Respiratory Syndrome (MERS) [5], both associated with high mortality and morbidity.

Noroviruses frequently cause acute gastroenteritis outbreaks globally, which is associated with heavy economic burden [6], as norovirus-induced gastroenteritis is particularly acute in the elderly and in young children. It is a highly resistant and infectious virus, with an infectious dose close to 20 virions and is easily transmitted through person-to-person contact [7]. The feline calicivirus strain F9 (FCV F9) is used to study the biology of norovirus [8], given the difficulties of growing human noroviruses in laboratory conditions.

The emerging of antimicrobial resistance is a problem for society [9]. Methicillin-resistant *S. aureus* (MRSA) is one of the pathogen strains causing the majority of hospital infections and effectively escapes the effects of antibacterial drugs [10]. MRSA causes different infections in the blood, heart, skin, soft tissue and bones. MRSA is also responsible for nosocomial infections. Treatment is often difficult, and currently, there is a need to develop new antimicrobials [11].

A bioprospecting of the bioactive molecules is reported from endophytic fungi, and the genus Curvularia and Bipolaris revealed interesting biological activities [12]. Some crude extracts of endophytes, isolated from medicinal plants of Sudan, have proven to be very rich and promising resources of bioactive compounds. For example, *Aspergillus* sp. associated with *Trigonella foenum-graecum* revealed powerful antioxidant activity [13,14]. *Byssochlamys spectabilis* and *Alternaria* sp. isolated from *Euphorbia prostrata* showed potent antiproliferative and antibacterial activities, respectively [15].

The genus Curvularia belongs to the family Pleosporaceae. Members of this genus are of widespread distribution in tropical and subtropical regions and are commonly isolated from a wide range of plant species as well as from soil. Many species are known to be plant pathogens [16]. Some researchers have demonstrated that crude extracts of some *Curvularia* species have interesting properties including antimicrobial, antioxidant, phytotoxicity and leishmanicidal activities [12,17,18,19].

Several natural products from different chemical classes were purified from the genus Curvularia: alkaloids such as curvulamine and curindolizine [20,21]; polyketides such as apralactone A, curvulide A and cochliomycin A [22,23,24]; quinones such as cynodontin and lunatin [25,26]; and terpenes such as zaragozic acid A [27].

In this study, an endophytic fungus was isolated from *Vernonia amygdalina*, and then the taxonomic characterization of the isolate was established. The ethyl acetate crude extract of the isolated endophyte was investigated for antiviral, antibacterial and antiproliferative activities. The crude extract was fractionated to afford pure compounds. Each fraction was analyzed by gas chromatography coupled to mass spectrometry (GC–MS). Structure elucidation of pure compounds was done using different spectroscopy techniques, and the biological activities were evaluated.

## 2. Materials and Methods

### 2.1. Chemicals

Ethyl acetate (≥99.9%), cyclohexane (≥99.9%), methanol (≥99.9%), acetic acid (≥99.9%), dichloromethane (≥99.9%), formic acid (≥99.9%), dextrose (≥99.9%), agar (≥99.9%), silica gel, tetramethylsilane (TMS), deuterated methanol, deuterated pyridine and deuterated dimethyl sulfoxide were purchased from Sigma-Aldrich Co. LLC, Steinheim am Albuch, Germany. All solvents used were LC analytical grade. The mixture of alkanes standard from C10 to C40 was purchased from Merck KGaA, Darmstadt, Germany.

### 2.2. Endophytic Fungus: Isolation and Taxonomic Characterization

Leaf and stem samples of *V. amygdalina* were collected from a plant species growing wild in Khartoum State, Sudan. Voucher specimen TN4010 was deposited at the herbarium of the Botany Department, Faculty of Sciences, University of Khartoum, Khartoum, Sudan. The protocol for the isolation of endophytic fungus from *V. amygdalina* plant materials was the same procedure described in the article of A. Khiralla et al. [15].

For the taxonomic characterization of the isolated fungus, the protocol is the same described by A. Khiralla et al. [15].

### 2.3. Cultivation of the Fungus and Extraction of the Metabolites

The fungus was cultivated and extracted according to the previous protocol [18] with minor modifications. Briefly, fungal strain was cultured on 750 Petri dishes (15 L). The Petri dishes were incubated at 28 ± 2 °C for 14 days. Then, the cultured plates were macerated using ethyl acetate for 24 h. The extraction was repeated three times [15]. The organic extract was stored at 4 °C after filtration and evaporation.

### 2.4. Biological Assays

#### 2.4.1. Cytotoxicity Tests: Cells, Media and Protocols

L132 (ATCC^®^ CCL5™) and CRFK (CCL-94™) cell lines were cultured in antibiotic-free Minimum Essential Medium Eagle (MEM, M4655, Sigma-Aldrich, St. Quentin Fallavier, France) complemented with 10% fetal bovine serum (FBS) (CVFSV F00-0U, Eurobio, Les Ulis, France).

Cytotoxicity of the crude extract was assessed in 96-well tissue culture plates. For this purpose, cells were seeded at 10^4^ cells per well in 96-well plates. The dry extract was dissolved in dimethylsulfoxide (DMSO), called DMSO-solubilized extract, or in sterile water called water-solubilized extract, then diluted in MEM medium complemented with 2% FBS. One hundred microliters of the diluted extract at increasing concentrations (2 to 256 µg/mL) were added to the cells monolayers 24 h following seeding. For the organic extract test, DMSO was used at 1% final concentration in order to avoid decrease in cell viability. The plates were incubated for 72 h at 37 °C in a 5% CO_2_ atmosphere. Viability of cells was evaluated with the MTT assay based on the reduction of the MTT by cellular metabolism into purple formazan in living cells [28].

After 72 h, the supernatants were replaced by 100 µL of MTT (0.5 mg/mL) (M2128, Sigma-Aldrich, St. Quentin Fallavier, France), prepared in MEM medium complemented with 2% FBS, and added to each well. The plates were incubated for 2 h at 37 °C. Finally, the wells were washed and formazan crystals were solubilized by the addition of 100 µL of DMSO (04474701, Biosolve, Dieuze, France). The plates were agitated until complete dissolution, and then the absorbance was read at 540 nm using a 96-well plate spectrophotometer (Multiskan GO, Thermo Scientific, Saint Herblain, France). Percentages of survival compared to control cells were calculated and the maximal concentration with no cytotoxic effect was determined using Microsoft Excel 2010 (Microsoft Corp., Redmond, WA, USA) and GraphPAD Prism v. 5 software (GraphPAD, San Diego, CA, USA).

#### 2.4.2. Antiviral Assay: Media, Viruses and Protocols

The infection medium, used for the antiviral assays, was the same as the growth medium, but 2% FBS was added instead. The human coronavirus HCoV 229E strain was propagated and quantified in L132 cells. CRFK cells were used for infection with the feline calicivirus FCV strain F9. Virus quantification was performed according to Reed and Muench’s method [29]. Briefly, the cells (10^4^ cells/well) were grown in 96-well tissue culture plates and incubated for 72 h in the presence of the HCoV 229E or FCV F9, at 33 °C and 37 °C, respectively, in a 5% CO_2_ atmosphere with serial 10-fold diluted virus suspensions in order to test multiplicity of infection (MOI), defined as the ratio of infectious virions to cells in a culture, between 0.0001 and 1. This protocol is similar to the article [30] The virus-induced cytopathogenic effect (CPE) was determined after 72 h of infection. By the method of Reed and Muench, the titers were counted as 50% Cell Culture Infectious Doses (CCID_50_)/mL. All virus stocks were stored at −80 °C until used.

The antiviral activity was evaluated by the reduction of the virus-induced cytopathogenic effect (CPE), characterized by different parameters such as rounding, vacuolation, syncytia formation and cell death of the cell monolayer. Different treatments were evaluated against each virus in 96-well tissue culture plates. One day before infection, cells were seeded at a concentration of 10^4^ cells/well. The next day, medium was removed and replaced by a mix consisting of diluted virus suspension and appropriate concentration of the extract. For each viral isolate (titers at least 10^6^ (CCID50)/mL), two sets of 1:10 serial dilutions were used from 1:10 (MOI 1) to 1:100,000 (MOI 0.0001), the first set without antiviral agent, and the second with a non-cytotoxic concentration of the extract. A blank without any cell, virus or extract was added. Eight wells were tested the same way for each assay, control or blank (*n* = 8). Plates were then incubated with HCoV 229E or FCV F9 at 33 °C and 37 °C, respectively, and were consequently checked for virus-induced CPE on days 1, 2 and 3 post-infection, using an inverted light microscope. The tests were read for determination of viral CPE when cell destruction in infected untreated cultures was at its maximum post-infection.

Consequently, estimation of the cytopathogenic effect was determined by the crystal violet (CV) assay, according to a previously described protocol with same adaptation [31]. Removal of cell culture medium, washing of cells with 1 × PBS ant then fixing with 3.7% formaldehyde (533,998, Sigma-Aldrich, St. Quentin Fallavier, France) for 5 min are doing in the CV uptake assay. Next, the cell monolayers were stained with 0.1% CV in PBS (C3886, Sigma-Aldrich, St. Quentin Fallavier, France) added to the same set of plates used to obtain the visual scores. After 30 min incubation at room temperature, the dye was removed, all the wells were washed two times with PBS, and uptaken CV was then solubilized with 100 µL methanol (525,102, Carlo Erba, Val-de-Reuil, France) per well and left for 5 min at room temperature. The color intensity of the dye uptake by the cells was measured with a 96-well plate spectrophotometer (Multiskan GO, Thermo Scientific, Saint Herblain, France) by reading optical density (OD) at 540 nm. The percentage of cytopathogenic effect (% CPE) was calculated for treated and non-treated infected wells according to the formula: ((OD_sample_ − mean OD_blank_)/mean OD_control_) × 100 where control was non-treated and non-infected cells. Antiviral activity was expressed as decrease of virus-induced CPE due to the treatment: % CPE (non-treated) − % CPE (extract-treated). Results are presented as the mean values obtained from at least two independent experiments.

##### Immunofluorescence Analysis (IFA)

An immunofluorescence analysis (IFA) was used to detect HCoV 229E and FCV F9 protein expression in infected host cells. Briefly, L132 and CRFK cells seeded on 96-well cell culture plates were grown for 24 h at 37 °C in 5% CO_2_ [30]. The cells were then infected with HCoV 229E and FCV F9 virus at MOI 1 and MOI 0.0001 and incubated for 24 h at 33 °C and 37 °C, respectively. At 24 h post-infections, 2% of paraformaldehyde was used to fix the cells and blocked in 5% bovine serum albumin (BSA) in 1% Triton-X-100 PBS. The infected cells were incubated with anti-HCoV 229E (FIPV3-70, St Cruz Biotechnology, Heidelberg, Germany) or anti-FCV F9 (FCV1-43, St Cruz Biotechnology) mouse primary antibody (1:500) for 1 h, washed three times with PBS and then incubated with 1:400-diluted FITC-labeled goat anti-mouse IgG (sc-2010, St Cruz Biotechnologies, Heidelberg, Germany) for 30 min. The identification of positive foci was done using fluorescence microscopy under an inverted fluorescence microscope (Zeiss, Marly-Le-Roi, France) after DAPI duplicate staining.

#### 2.4.3. Antibacterial Assay: Cells, Media and Protocols

In this study, eighteen standard strains of bacteria were used. Gram-negative bacteria: *Pseudomonas aeruginosa* (CIP82118), *Salmonella enterica* subspecies *enterica* sérovar Abony, *Escherichia coli* (ATCC 8739). Gram-positive bacteria: *Staphylococcus aureus* (ATCC 6538), MRSA, *S. arlettae*, *S. capitis*, *S. hominis*, *S. auricularis*, *S. epidermidis*, *S. haemolyticus*, *S. xylosus*, *S. lugdunensis*, *S. sciuri*, *Enterococcus faecalis*, *E. faecium*, *Bacillus cereus*, *Kytococcus sedentarius* [14].

Information for the media and protocols are in the article by A. Khiralla et al. [15].

#### 2.4.4. Antiproliferative Activity: Cells, Media and Protocol

The cells used for the determination of antiproliferative activities were the human colon adenocarcinoma (HT29 and HCT116) and human breast adenocarcinoma (MCF7). The media used for the cultivation and protocols are described in the article by Khiralla A. et al. [15]. For antiproliferative activities, the respective IC_50_ value was calculated from results obtained from quadruplicate determination of two independent experiments (*n* = 8). IC_50_ value was expressed as µg/mL of extract diluted in DMSO.

### 2.5. Analytical and Spectroscopic Analysis

For identification and characterization of metabolites, the following were used: a thin-layer chromatograph (TLC GF_254_ plates (Merck), an infrared (IR) spectrometer (Perkin-Elmer model 1650 FTIR), a Bruker Avance III 400 M Hz spectrometer, and a gas chromatography system coupled with a mass spectrometer (GC–MS, QP2010-Shimadzu equipment). The column used was an SLB5 column DB-5 ms, the procedure of which was the same as that described in our previous works [32,33]. The following were also used: a liquid chromatography system (U3000-Dionex apparatus) coupled with a mass spectrometer (Bruker Daltonics micrOTOF-Q^TM^); a high-resolution liquid chromatography system (HPLC, Merck Hitachi Lachrom) for analytical analysis, in which the analytical column used was a C18 ODS Hypersil^TM^ 5 µM 250 × 4.6 mm column (Thermo Scientific, USA); a preparative HPLC (Gilson), for which the semi-preparative column used was ODS Hypersil^TM^ C18 250x10mm (ThermoScientific, USA). For all technical characteristics of equipment, refer to Elmi et al.’s [32] and A. Khiralla’s work [14].

### 2.6. Purification and Identification of Metabolites from C. papendorfii Crude Extract

The dark brown ethyl acetate crude extract of *C. papendorfii* contained a white precipitate. The precipitate was physically separated, washed with ethyl acetate and then subjected to fractionation and analyzed. At the end, we recovered 52 mg. The first analysis was realized by TLC. The mobile phase for TLC was made of 7/3/0.1 (*v/v/v*) ethyl acetate/cyclohexane/glacial acetic acid. The plates were observed under UV254 and UV365 nm and then sprayed with sulfuric acid reagent. The precipitate was injected to GC–MS, LC–MS and HPLC. The precipitate (25 mg) was dissolved in 1 mL of a mixture of methanol and water (0.9 mL and 0.1 mL respectively) and purified with semipreparative HPLC, using a linear gradient consisting to a mixture of methanol with 2% formic acid and water with 2% formic acid to give compound **1** (7 mg).

The ethyl acetate fraction was purified with an open column of silica gel, with a gradient of cyclohexane/ethyl acetate, (9:1 to 2:8 (*v/v*)) and then ethyl acetate/methanol/acetic acid (8:1:1 (*v/v/v*)). At the end, the column was washed with methanol. The tubes were collected using TLC profile. Ten fractions (F1, F2, F3, F4, F5, F6, F7, F8, F9 and F10) were obtained, and all were evaporated under a vacuum. In the fraction F6, a crystalline solid could be observed, compound **2** (12 mg). Fraction F10 was purified using an open column (using a mobile phase constituted by EtOAc/MeOH/acetic acid). The purification led to six subfractions called F10.A, F10.B, F10.C, F10.D, F10.E and F10.F. All subfractions were analyzed by TLC and GC–MS. A total of 23 compounds were determined by GC–MS. The identification of the chemical compounds was obtained by comparison of mass spectra with the spectra present in the NIST (National Institute of Standards and Technology) library. The retention index was calculated using alkane standard mixture (C10–C40) under the same operating conditions.

## 3. Results and Discussion

### 3.1. Isolation and Taxonomic Characterization of the Fungal Strain

**Figure 1:**
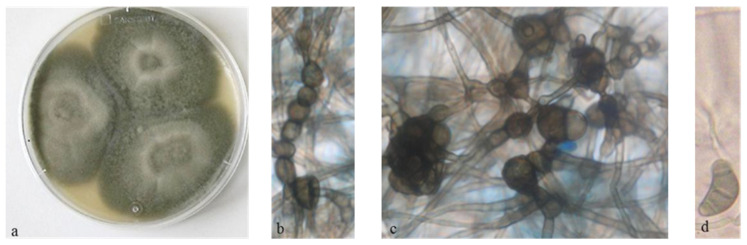
*Curvularia papendorfii* fungus; (**a**): culture on Potato dextrose agar (PDA) plate; (**b**,**c**): chlamydospores ×400; (**d**): conidia ×400 [14].

The endophytic fungus was isolated from both leaf and stem samples of *V. amygdalina* plant, which showed a colonization frequency (CF) of 90%. High CF was also recorded on previous studies on fungal endophytes communities such as in Puerto Rico and Sudan [15,34]. ITS sequences were deposited in GenBank and then compared using a BLAST search [32]. The isolated fungus was identified as *C. papendorfii* with 99% identity (Genbank number KR673909) (Figure 1).

In this study, for the first time, *C. papendorfii* was reported as a fungal endophyte in *V. amygdalina*, collected in Sudan, although most *Curvularia* species occur as tropical and subtropical plant pathogens [12,35]. The genus Curvularia was also recovered from several plants as endophytes [36,37], as endolichens [38] and as marine-derived fungus [22,23,39].

### 3.2. Screening of Biological Activities of C. papendorfii Crude Extract

#### 3.2.1. Cytotoxic Effects

**Figure 2:**
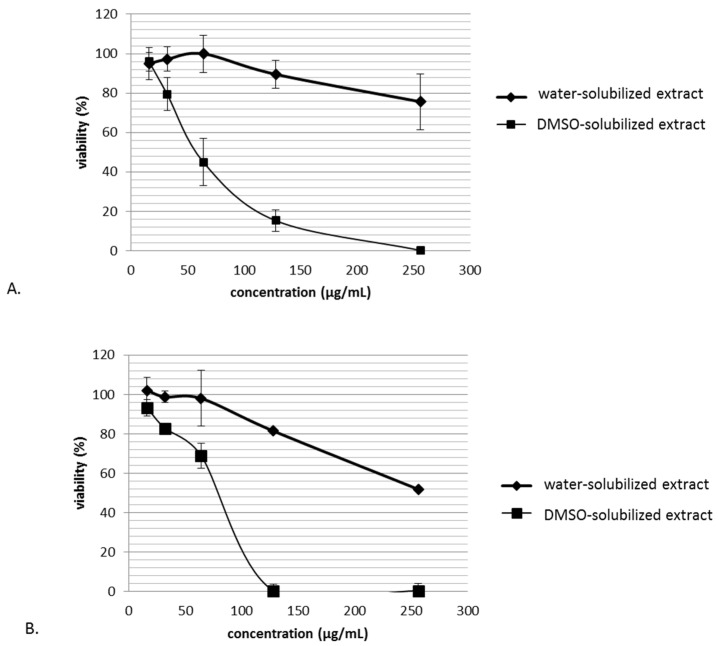
Cytotoxicity of *C. papendorfii* DMSO-solubilized extract and water-solubilized extract after 72 h treatment on L132 (**A**) and CRFK (**B**) cells. Data show mean for *n* = 3 independently performed experiments. Bars indicate standard deviations.

In order to exclude non-specific activities of the extract, cytotoxic effects had to be evaluated on cell lines L132 (A) and CRFK (B) cells, and the maximum non-toxic concentration for the cells had been determined (Figure 2).

The ethyl acetate crude extract of *C. papendorfii* was dissolved at 25 mg/mL in dimethylsulfoxide (DMSO). This extract is called DMSO-solubilized extract. Water was also tested to solubilize the extract and evaluate the effect of extract dissolution on cytotoxic effect. This extract is called water-solubilized extract. L132 and CRFK cell lines were treated with different concentrations of extract. The extract seemed to be more toxic when dissolved in DMSO for both cell lines. The CRFK cell line was more sensitive to the DMSO-solubilized extract than the L132 cell line. According to these results, the concentration 16 µg/mL was the dose chosen for antiviral tests. The low toxicity of water-solubilized extract (10.5 ± 6.9%) allowed application in the antiviral treatment at 128 µg/mL.

#### 3.2.2. Antiviral Activity of Crude Extract

For both viruses, HCoV and noroviruses, there is no vaccine or antivirals drugs for the prevention and the treatment of infection. In the absence of curative antiviral strategies, the bioactive molecules of fungal origin are particularly important as novel drug candidates.

The impact of the crude extract on viral infection was evaluated by the reduction of virus-induced cytopathogenic effect. L132 and CRFK were infected by the human coronavirus and the feline calicivirus, respectively, in the presence or absence of the extract for 72 h. The use of L132 cell line allowed producing a high level of viral titers and obtaining high sensitivity in antiviral evaluation, as previously demonstrated [40], notwithstanding the fact that there has been a contamination with HeLa cells as described recently by ATCC. This cell line is still made available by ATCC as a reference cell line, and it is currently used as a host cell line for HCoV 229E, as it allows one to obtain reproducible results in terms of antiviral tests. Given that this study does not involve or require specific organ or tissue of presumptive origin and that human coronaviruses have wide tissue and cellular tropism, the results of the virus production and assays remain uncompromised in these conditions.

**Figure 3:**
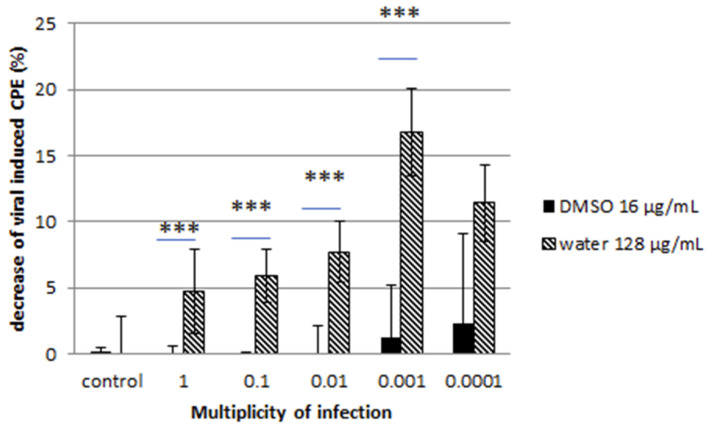
Effect of *C. papendorfii* DMSO-solubilized extract and water-solubilized extract on the infection of L132 cells by the human coronavirus 229E. Results are presented as a decrease of virus-induced cytopathogenic effect (CPE) at 72 h post-infection, calculated as % CPE (non-treated) − % CPE (extract-treated). Data are shown for at least two independent experiments. *T-test* statistical analysis was performed with GraphPAD Prism 5 software (*** means *p* < 0.005).

**Figure 4:**
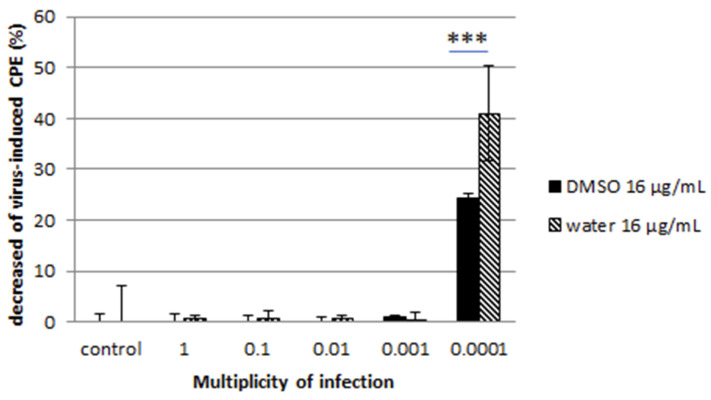
Effect of *C. papendorfii* DMSO-solubilized extract and water-solubilized extract on the infection of CRFK cells by the feline calicivirus FCV F9. Results are presented as a decrease of virus-induced CPE, at 72 h post-infection, calculated as % CPE (non-treated) − % CPE (extract-treated). Data are shown for at least two independent experiments. Bars indicate standard deviations. *T-test* statistical analysis was performed with GraphPAD Prism 5 software (*** means *p* < 0.005).

Cell monolayers were then stained with CV, and the percentage of CPE was calculated. A decrease of CPE was reported for both viruses at low multiplicity of infection ratio (Figure 3 and Figure 4).

**Figure 5:**
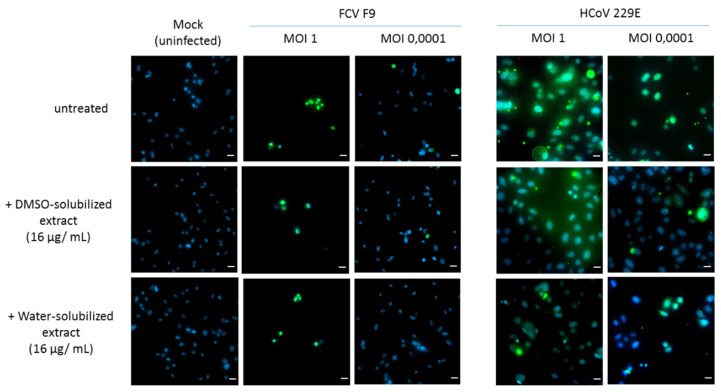
Immunofluorescence staining of FCV F9- and human coronavirus (HCoV) 229E-infected cells. CRFK and L132 cells were infected with FCV F9 and HCoV 229E, respectively, for 24 h and treated with DMSO-solubilized extract and water-solubilized extract (16 µg/mL) prior to fixation with methanol. The infected cells were then incubated with anti-FCV F9 and anti-HCoV 229E antibodies, then stained with goat anti-mouse FITC secondary antibody and treated with DAPI as counterstain. The photos were obtained at 40× magnification power. Scale bars: 20 µm.

The water-solubilized extract was more effective than the DMSO-solubilized extract. The reduction reached 40% for the water-solubilized extract at lower MOI for the feline calicivirus while it was more moderate for the coronavirus. These observations were confirmed by the immunofluorescence assay, where a clear reduction of HCoV- and FCV F9-positive host cells was noted (Figure 5).

Here we report an important antiviral effect of *C. papendorfii* extracts on two viral pathogens—the human coronavirus HCoV 229E and a norovirus surrogate, the feline coronavirus FCV F9.

Our exploration of the antiviral properties of DMSO-solubilized extract and water-solubilized extract of the fungal endophyte *C. papendorfii* indicated an important antiviral effect on enveloped viruses such as HCoV 229E at all MOI tested, even at MOI as high as MOI 1. This effect is particularly interesting in the case of water-solubilized extract with a reduction of the virus-induced cytopathogenic effect over 15% at MOI 0.001. The impact on virus-induced CPE by water-solubilized extract was also important in the case of non-enveloped viruses, represented in our study by FCV F9, with close to 40% reduction. This effect was observed exclusively at lower MOI, such as MOI 0.0001, which can be explained by the important infectivity and resistance of the virions. Indeed, non-enveloped viruses are much more stable and may stay active in wastewaters and on environmental surfaces for several months [41,42]. On the other hand, enveloped viruses are less stable and more prone to degradation. Enveloped viruses of the respiratory tract like influenza virus and coronavirus can persist on surfaces only for several days [43]. We observed that incubation with the plant extract during infection impaired the productive replication of both viruses in a MOI-dependent manner. The results obtained suggest that *C. papendorfii* antiviral activity might be partially due to a direct interaction of the compounds in the extract with the viral envelope, given the effect on HCoV. However, the reduction of the FCV F9 infection at lower MOI is a piece of evidence in favor of extract-induced interference with intracellular virus-induced macromolecular synthesis, thus hampering the viral replication. The predominant antiviral effect of water-solubilized extract indicated that polar biomolecules may be in the center of the antiviral activity, which is in accordance with the existing literature [44].

Our results allowed us to describe for the first time the capacity of *C. papendorfii* extracts to inhibit viral infection. Previous study has shown that crude extracts of one endophytic *Curvularia* species isolated from Garcinia plants [45] present antimycobacterial properties but no antiviral activity using herpes simplex virus (HSV-1) infection, even at the highest concentration (50 µg/mL) tested [45].

#### 3.2.3. Antibacterial Activity of Crude Extract

A preliminary antibacterial screening was performed for ethyl acetate crude extract of *C. papendorfii* using agar disk diffusion method against 18 Gram-positive and Gram-negative bacterial strains. The results obtained indicated that the ethyl acetate crude extract of *C. papendorfii* had an effective antimicrobial activity against most Gram-positive bacteria, and no effect was observed against three Gram-negative bacterial strains [14].

The crude extract exhibited a maximum inhibition zone of 13 mm against MRSA and *S. aureus.* The inhibition zones were compared with the positive control, nitrofurantoin (27 mm), while 12 mm inhibition zone was reported against *S*. *epidermidis* and *S*. *capitis*; 10 mm against *S. lentus*, *S. warneri*, *S. sciuri*, *S. xylosus*, *S. haemolyticus* and *S. lugdunensis*; and 9 mm against *E. faecalis*, *K. sedentarius* and *S. arlettae*. In contrast, no inhibitory effect against *E. faecium*, *B. cereus*, *P. aeruginosa*, *E. coli* or *S. abony* was observed (Table 1).

For *Staphylococcus aureus* and MRSA, the inhibition zone of 27 mm was determined with Nitrofurantoin (100 µg). Nitrofurantoin was used as a positive control when the inhibition zone was ≥ 13 mm [46].

The antibacterial activities of crude extract of *C. papendorfii* were confirmed by the broth dilution method, and the MIC values were 312 µg/mL for *S. aureus* as well as for MRSA.

However, several studies revealed that *Curvularia* sp. crude extracts have promising antibacterial activities. The extract of *C. lunata* showed antibacterial activities against *S. aureus* and *S. typhi* [17], and the ethyl acetate crude extract of *C. tuberculata* has shown antibacterial activities against *S. aureus*, *E. coli* and *P. aeruginosa* [39]. Moderate activities of extract of *Curvularia* B34 against *Bacillus subtilis, Listeria monocytogenes* and *Salmonella* bacteria have been observed [37]. *C. hawaiiensis*, an endophytic fungus isolated from *Calotropis procera,* showed antibacterial activity against *Serratia marcescens* [47]. The extract of endophytic fungus *Curvularia* sp. T12 isolated from *Rauwolfia macrophylla* had antibacterial activity against *E. coli*, *Micrococcus luteus*, *Pseudomonas agarici* and *S. warneri* [48].

#### 3.2.4. Antiproliferative Activity of Crude Extract

Antiproliferative activity of ethyl acetate crude extract of *C. papendorfii* was evaluated using human cancer cell lines. The results obtained showed that the IC_50_ value was 21.5 ± 5.9 µg/mL for human breast adenocarcinoma (MCF7), and the IC_50_ values were higher than 100 µg/mL for the two cell lines of human colon adenocarcinoma (HT29 and HCT116). Response–dose curves established from results obtained by MTT assays after MCF7, HT29 and HCT116 cells exposure of *C. papendorfii* are presented in Figure S1.

In our previous work [15], the stem extract of *V. amygdalina*, the host plant of endophytic *C. papendorfii*, also showed cytotoxic effect against HT29 and MCF7 (IC_50_ values of 15.3 ± 3.6 and 49.6 ± 4.4 µg/mL respectively), while leaf extract had strong cytotoxicity (IC_50_ value of 5.6 ± 0.4 µg/mL) against HCT116 and moderate cytotoxicity (IC_50_ value of 27.5 ± 5.7 and 31.9±5.1 µg/mL) against MCF7 and HT29, respectively.

The cytotoxicity of ethyl acetate crude extract of *C. papendorfii* for MCF7 cell line may be in relationship with the chemical constituents as has been shown with polyketides extracted from marine-derived fungus *Curvularia* sp. [23].

### 3.3. Phytochemical Analysis

After purification, two pure compounds were identified, using mono and bidimensional Nuclear Magnetic Resonance (NMR), Mass Spectrometry (MS) and Infrared (IR) analysis. One is a new compound, never described in the literature and given the trial name kheiric acid (compound **1**), and the second one is a known structure, mannitol (compound **2**). The other compounds were identified by GC–MS analysis and compared with literature bibliography.

For the characterization of compound **1**, an absorption band on the IR spectrum at 2914 cm^−1^ is typical of a carboxylic group. An absorption band at 1714 cm^−1^ is predictive of a C=O group. The weak absorption band at 1666 cm^−1^ indicates alkenes functionality. A strong absorption band at 3383 cm^−1^ indicates OH groups (Figure S3).

The high-resolution electrospray ionization mass spectrometry (HRESIMS), positive mode, gave molecular ion peak of *m/z* 573.3781 [M + Na]^+^ (Figure S4). The HRESIMS, negative mode, gave a molecular ion peak of *m/z* 549.3793 [M − H]^−^. The possible molecular formula is C_32_H_54_O_7_. The index of hydrogen deficiency (IHD) reveals an unsaturation index of 6.

Referring to the ^13^C NMR spectrum and the ^1^H NMR spectrum, 32 carbon signals and 48 protons are evident. By *J*-modulated spin-echo (*J*-mod) spectrum, it is possible to observe six CH_3_, six CH_2_, and seventeen CH signals in addition to three quaternary carbon atoms. By ^1^H and ^13^C NMR spectrum in methanol *d_4_* and consequently by observation of COSY and HMBC spectra, it may be deduced that six signals are indicative of methyl groups—22.9 (C-30), 20.7 (C-32), 20.3 (C-31), 17.9 (C-27), 13.5 (C-28), 11.7 (C-26) ppm; ten carbon signals are indicative of olefinic carbons—140.0 (C-19), 139.0 (C-16), 136.3 (C-12), 136.0 (C-9), 135.9 (C-18), 135.6 (C-4), 134.1 (C-8), 129.9 (C-17), 128.9 (C-5), 128.8 (C-13) ppm—and five peaks signify carbons with a carbon–oxygen single bond: 83.7 (C-15), 73.4 (C-11), 73.7 (C-7), 70.3 (C-3), 61.2 (C-29) ppm. The last one is characteristic of primary alcohol. The others are secondary hydroxyl functions. The deshielding of the carbon C-1 at 175.3 ppm suggests the presence of carbonyl function of carboxylic acid. The methylene group H-2 and H-2′ at 2.47 ppm is correlated in HMBC with acid carbon C-1 (175.3 ppm). The methylene group at 2.47 ppm is also correlated with two other signals in HMBC: C-3 (70.3 ppm) and C-4 (135.6 ppm). An H-C coupling was observed between H-4 and C-5, corresponding to one double bond. In the ^1^H NMR spectra, we observed a section comprising two repeated 4 carbon units: a methylene (H-6 and H-10), a hydroxymethine (H-7 and H-11) and a disubstituted double bond (H-8/H-9 and H-12/H-13). In this section a set of twinned NMR signals are present. The data suggest the presence of 3 double-bonded carbons with a coupling constant of 15 Hz (H-4/H-5), (H-8/H-9), (H-12/H-13) that confirm the configuration trans. Two trisubstituted unsaturated bonds were observed, C-16/C-17 and C-18/C-19. One of the methyl groups is located on the second trisubstituted unsaturated bonds (d, 1.77 ppm, CH3-28 at C-16). By COSY, it was possible to observe the correlation between CH3-28 and H-17. The primary alcohol CH_2_OH (AB system for H-29 and H-29′ at C-18) is located on one of the two trisubstituted unsaturated bonds. The NMR characterization was also reported in A. Khiralla’s work [14].

The NMR characterization was also realized using pyridine *d_5_* as solvent due to signal overlap and the high degree of functionalization. By the analysis, the presence may be deduced of 32 signals of carbons in the ^13^C NMR spectrum in pyridine *d_5_* and 53 protons in the ^1^H NMR. From ^1^H NMR spectrum of compound **1** in pyridine *d_5,_* it was possible to observe a good separation in the region of double bonds (6.4 ppm–5.9 ppm). We can clearly determine the presence of five methyl groups (doublets respectively a 2.17, 1.16, 1.06, 0.91, 0.84 ppm) and one other CH_3_ (triplet at 0.85 ppm). Four methylene protons were distinguished at 2.58 ppm and 2.64 ppm. All the data are shown in Table 2 as reported in [14].

The analysis and the original NMR spectra are provided in supporting information (Figures S5–S13).

The presence of five double bonds and one insaturation of carboxylic acid suggests that the structure is a long chain. The compound **1** is a polyfunctionalized long-chain carboxylic acid.

**Figure 6:**
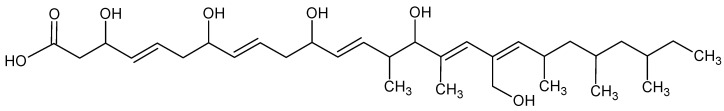
Chemical structure of the new polyhydroxyacid, kheiric acid (compound **1**).

The isolated compound was identified as 3,7,11,15-tetrahydroxy-18-hydroxymethyl-14,16,20,22,24-pentamethyl-hexacosa 4E,8E,12E,16,18-pentaenoic acid [14], which is named kheiric acid (Figure 6). Taking into account all the spectroscopic results, the structure is a new compound and has never been described in the literature.

**Figure 7:**
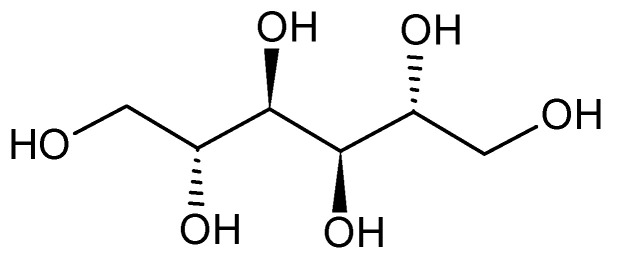
Chemical structure of mannitol (compound **2**).

The analysis of the fraction 6 of *C. papendorfii* endophytic fungus extract revealed the presence of mannitol (compound **2**). A clear crystalline solid (5 mg) was obtained. HRESIMS (positive mode) *m/z* 205.0691 [M + Na]^+^ (calcd. for C6H14O6Na, 205.0688) (Figure S15). ^1^H NMR (400 M Hz, DMSO *d_6_*): *δ* (ppm) 4.42 (d, *J* = 5.4 Hz), 4.34 (t, *J* = 5.8 Hz), 4.15 (d, *J* = 7.1 Hz), 3.63 (m) 3.58 (m), 3.49 (m), 3.39 (m). ^13^C NMR (100 M Hz, DMSO *d_6_*): *δ* (ppm) 71.4, 69.8, 63.9. The ^1^H and ^13^C spectra and bidimensional NMR were compared with original standard and with the literature (see supporting information, Figures S16–S20). The chemical structure is presented in Figure 7. This compound is a polyol extracted naturally from plants, bacteria and fungi [49,50]. Mannitol is a chemical metabolite used currently in the field of food and drugs [51].

The GC–MS analysis of the fractions and subfractions led to the identification of twenty-two compounds (Table 3).

The GC–MS analysis of the fraction F1 reveals the presence of four compounds: 2,4-di-tert-butylphenol, 1-octadecene, undec-10-ynoic acid and benzenepropanoic acid, 3,5-bis(1,1-dimethylethyl)-4-hydroxy-, octadecyl ester.

Fraction F3 contained a mixture of four fatty acids identified as methyl palmitoleate, methyl petroselinate, methyl stearate and methyl linolelaidate.

The identifications of five compounds were observed in fraction F10.C: 1b,5,5,6a-tetramethyl-octahydro-1-oxa-cyclopropa[a]inden-6-one, 4-oxo-β-isodamascol, oleic acid amide, 1,2-octadecanediol and cyclopentadecanol.

In fraction F10.D were identified four compounds: dicyclohexane, 3-dodecyl-2,5-furandione, methyl elaidate and 2,4-di-tert-butylphenol. This last compound was also found in the fraction F1.

In fraction F10.E, the following compounds were identified: n-dodecenylsuccinic anhydride, 9-eicosene, the methyl ester of 11,14-eicosadienoic acid, the methyl ester oleic acid and two compounds previously observed, the 2,4-di-tert-butylphenol present in the fractions F1 and F10.D and 1-octadecene, also present in the fraction F1.

Analysis of the fraction precipitate reveals three compounds: ascaridole, 1b,5,5,6a-tetramethyl-octahydro-1-oxa-cyclopropa[a]inden-6-one and 1-eicosanol.

**Figure 8:**
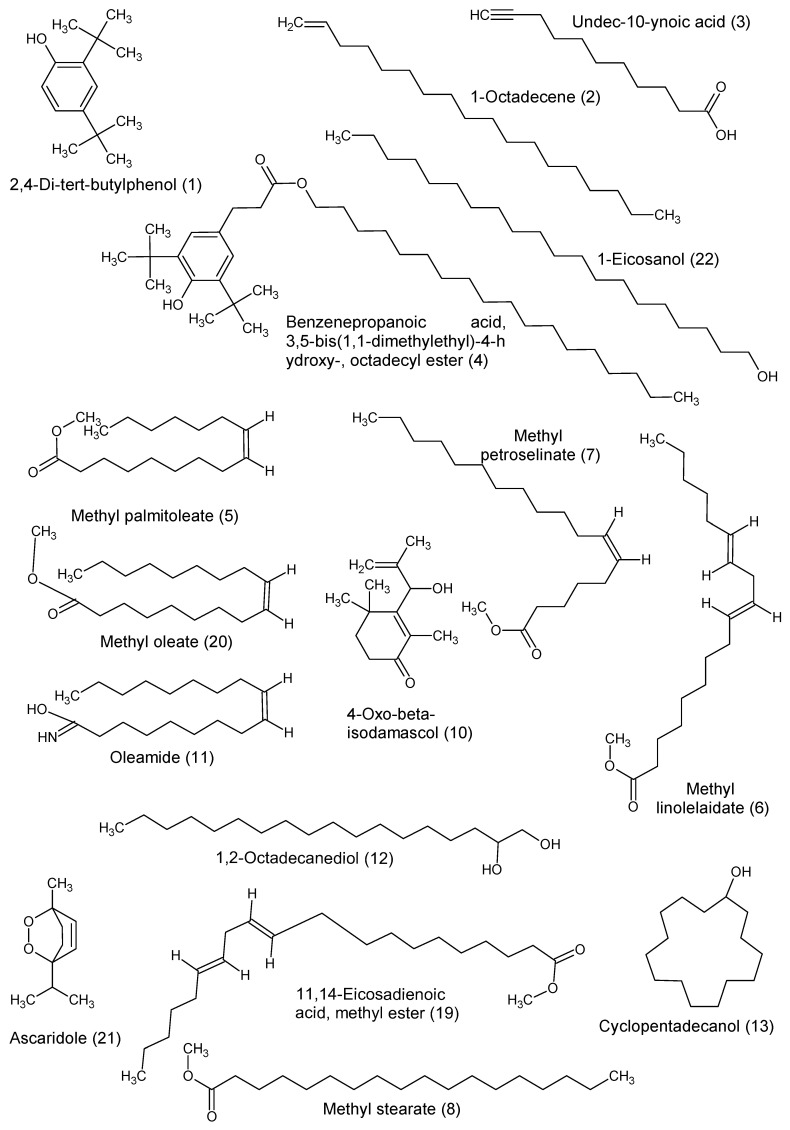

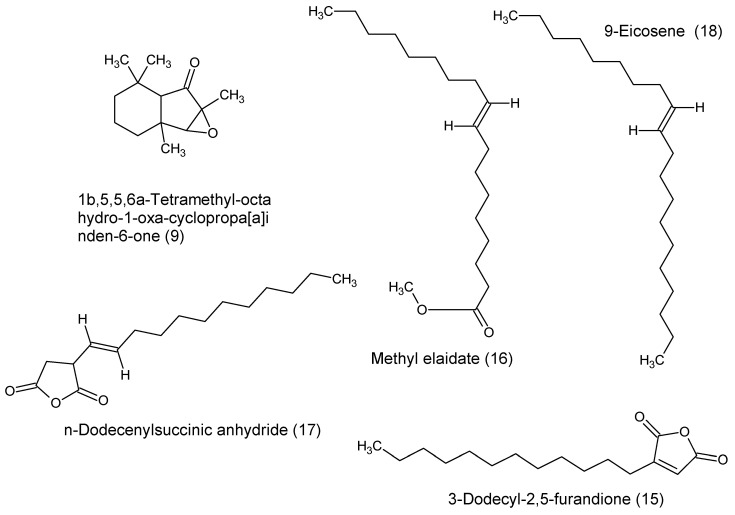
Chemical structures identified by GC–MS.

The chemical structures are presented in Figure 8.

### 3.4. Biological Activities of Identified Compounds and Kheiric Acid

Some chemical compounds identified by GC–MS have shown some interesting biological properties. For example, undec-10-ynoic acid is an inhibitor of cytochrome P450 4A1 [53], and fatty acids such as methyl palmitoleate, methyl linolelaidate and methyl stearate showed antimicrobial activities against *Streptococcus mutans* and some human fungi pathogens [54]. The methyl petroselinate has antioxidant activity [55]. Ascaridol, a bicyclic monoterpenoid showed cytotoxicity against MDA MB-231 breast cancer [56]. The presence of these bioactive compounds in the crude extract of *Curvularia papendorfii* can justify its interesting biological activities as antiviral, antibacterial and antiproliferative properties.

In Table 4, the biological activities of endophytic fungus crude extract and pure kheiric acid are presented. Kheiric acid revealed moderate activity (MIC = 62.5 µg/mL) against Gram-positive MRSA and *S. aureus*. This result is promising because the isolated compound **1** has a better value than the crude extract, which showed an MIC of 312 µg/mL. No antiviral and antiproliferative activities of this compound against MCF7, HT29 or HCT116 cell lines were observed. This loss of activity could be related to the fractionation.

Some analogs of polyhydroxyacid kheiric acid were isolated from different fungal genera. Phomenoic acid (C_34_H_58_O_8_) was purified from the mycelium of *Phoma lingam*; this compound showed moderate antifungal and antibacterial in vitro, in particular against *Candida albicans* [57,58]. Arthrinic acid (C_32_H_54_O_9_) was isolated from the crude extract of the fungus *Arthrinium phaeospermum*. Arthrinic acid showed antifungal activity against *Botrytis cinerea*, *Rhizopus stolonifera* and *Diplodia pinea* as pathogens in horticulture [59].

## 4. Conclusions

*C. papendorfii* is an endophytic fungus associated with *V. amygdalina,* a Sudanese medicinal plant. In this study, the ethyl acetate crude extract of *C. papendorfii* was extensively studied. The reduction of 40% for water-solubilized extracts at a lower MOI of 0.0001 is an important result against the feline calicivirus FCV F9 because caliciviruses are generally much more resistant than coronaviruses. Until now, no treatment is present for this virus. In addition, this extract revealed interesting antiproliferative effects against human breast carcinoma MCF7 cell line (IC_50_ = 21.5 ± 5.9 µg/mL) and an MIC value of 312 µg/mL against meticillin-resistant *Staphylococcus aureus* (MRSA). A total of twenty-four chemical structures were identified. The major compound isolated from the ethyl acetate crude extract of *C. papendorfii* was a new compound, a polyhydroxyacid, kheiric acid (3,7,11,15-tetrahydroxy-18-hydroxymethyl-14,16,20,22,24-pentamethyl-hexacosa 4E,8E,12E,16,18-pentaenoic acid). This compound revealed moderate antibacterial activity against MRSA and *S. aureus* with MIC value 62.5 µg/mL. Once more, these results confirm the importance of endophytic fungi as a source of new biomolecules.

## Figures and Tables

**Table 1:** Antimicrobial activity of crude extract of *C. papendorfii* isolated from *V. amygdalina* against several Gram-positive and Gram-negative bacterial strains in an agar diffusion assay.

Bacterial Strains	Inhibition Zone (mm) with Ethyl Acetate Extract
*P. aeruginosa*	6
*E. coli*	6
*Salmonella* Abony	6
*Staphylococcus aureus*	13
MRSA	13
*S. arlettae*	9
*S. lentus*	10
*S. epidermidis*	12
*S. haemolyticus*	10
*S. xylosus*	10
*S. sciuri*	10
*S. warneri*	10
*S. capitis*	12
*S*. *lugdunensis*	10
*E. faecalis*	9
*E. faecium*	6
*B. cereus*	6
*K. sedentarius*	9

The inhibition zone was measured in mm and derived from experiments in triplicates. Standard deviation value is ± 0.58 for all bacteria.

**Table 2:** NMR data of pure compound **1**, kheiric acid.

Position	Methanol *d_4_*		Pyridine *d_5_*	
	^13^C *δ*_C_	^1^H *δ*_H_ (*J* in Hz)	^13^C *δ*_C_	^1^H *δ*_H_ (*J* in Hz)
C-1	175.3	-	175.0	-
C-2	43.5	2.47 m (2H, H-2, H-2′)	44.6	3.05 dd (1H, *J* = 15 Hz, *J* = 8.5 Hz, H-2′) 2.96 dd (1H, *J* = 15 Hz, *J* = 5 Hz, H-2)
C-3	70.3	4.47 m (1H, H-3)	69.9	5.18 m (1H, H-3)
C-4	135.6	5.59 dd (1H, H-4, *J* = 6.6 Hz, 18 Hz)	136.5	6.10 dd (1H, H-4, *J* = 6 Hz, 15 Hz)
C-5	128.9	5.73 dd (1H, H-5, *J* = 7 Hz, 15 Hz)	128.0	6.32 dd (1H, H-5, *J* = 7 Hz, 15 Hz)
C-6	41.4	2.26 m (2H, H-6, H-6′)	42.0	2.58 m (2H, H-6, H-6′)
C-7	73.7	4.05 m (1H, H-7)	72.7	4.52 m (1H, H-7)
C-8	134.1	5.48 dd (1H, H-8, *J* = 7 Hz, 15 Hz)	137.0	5.97 dd (1H, H-8, *J* = 6 Hz, 15 Hz)
C-9	136.0	5.64 dd (1H, H-9, *J* = 7 Hz, 15 Hz)	127.9	6.21 dd (1H, H-9, *J* =7.6 Hz, 15 Hz)
C-10	41.5	2.23 m (2H, H-10, H-10′)	42.1	2.64 m (2H, H-10, H-10′)
C-11	73.4	4.03 m (1H, H-11)	72.9	4.51 m (1H, H-11)
C-12	136.3	5.54 dd (1H, H-12, *J* = 7 Hz, 18 Hz)	134.8	5.98 dd (1H, H-12, *J* = 6 Hz, 15 Hz)
C-13	128.8	5.70 dd (1H, H-13, *J* = 7 Hz, 15 Hz)	134.5	6.27 dd (1H, H-13, *J* = 7.6 Hz, 15 Hz)
C-14	41.6	2.35 m (1H, H-14)	41.35	2.72 m (1H, H-14)
C-15	83.7	3.72 d (1H, H-15, *J* = 8.6 Hz)	82.8	4.18 d (1H, *J* = 8 Hz, H-15)
C-16	139.0	-	139.5	-
C-17	129.9	5.92 s (1H, H-17)	128.9	6.51 bs (1H, H-17)
C-18	135.9	-	137.1	-
C-19	140.0	5.14 d (1H, H-19, *J* = 10 Hz)	138.3	5.40 d (1H, *J* = 10 Hz, H-19)
C-20	31.4	2.70 m (1H, H-20)	30.7	2.91 m (1H, H-20)
C-21	46.7	1.30 m (1H, H-21) 1.05 m (1H, H-21′)	45.9	1.42 m (1H, H-21′) 1.07 m (1H, H-21)
C-22	32.9	1.43 m (1H, H-22)	32.0	1.43 m (1H, H-22)
C-23	46.8	1.20 m (1H, H-23) 0.95 m (1H, H-23′)	45.9	1.22 m (1H, H-23′) 0.95 m (1H, H-23)
C-24	29.5	1.52 m (1H, H-24)	28.7	1.66 m (1H, H-24)
C-25	30.5	1.09 m (1H, H-25) 1.39 m (1H, H-25′)	29.9	1.29 m (1H, H-25′) 1.07 m (1H, H-25)
C-26	11.7	0.86 t (3H, CH3, H-26, *J* = 7 Hz)	11.7	0.85 t (3H, CH_3_, *J* = 7 Hz, C-26)
C-27	17.9	0.89 d (3H, CH3, H-27, *J* = 7 Hz)	18.4	1.16 d (3H, CH_3_, *J* = 6.6 Hz, C-27)
C-28	13.5	1.77 d (3H, CH3, H-28, *J* = 1.3 Hz)	14.2	2.17 d (3H, CH_3_, *J* = 0.9 Hz, C-28)
C-29	61.2	4.16 AB system, d (1H, H-29, *J* = 12 Hz) 4.16 AB system, d (1H, H-29′, *J* = 12 Hz)	61.0	4.60 d (1H, *J* AB = 12 Hz, H-29′) 4.58 d (1H, *J* AB = 12 Hz, H-29)
C-30	22.9	0.99 d (3H, CH3, H-30, *J* = 6.6 Hz)	23.1	1.06 d (3H, CH_3_, *J* = 6.6 Hz, C-30)
C-31	20.3	0.84 d (3H, CH3, H-31, *J* = 6.6 Hz)	20.1	0.84 d (3H, CH_3_, *J* = 6 Hz, C-31)
C-32	20.7	0.87 d (3H, CH3, H-32, *J* = 6.6 Hz)	20.8	0.91 d (3H, CH_3_, *J* = 6.6 Hz, C-32)
			-	4.98 broad m (5H, 5-OH)

Chemical shifts (*δ*) are in parts per million (ppm); coupling constants (*J*) are in hertz ( Hz).

**Table 3:** Metabolites identified using GC–MS analysis.

Fraction Name	Compound Number	Compound Name	Formula	Molecular Weight	Calculated Retention Index	Retention Time (min)	Similarity (%)
F1	1	2,4-Di-tert-butylphenol	C14H22O	206	1447	14.21	96
F1	2	1-Octadecene	C18H36	252	1730	17.610	96
F1	3	Undec-10-ynoic acid	C11H18O2	182	2262	22.797	80
F1	4	Benzenepropanoic acid, 3,5-bis(1,1-dimethylethyl)-4-hydroxy-, octadecyl ester	C35H62O3	530	3598	32.063	87
F3	5	Methyl palmitoleate	C17H34O2	270	1986	20.257	94
F3	6	Methyl linolelaidate	C19H34O2	294	2159	21.887	94
F3	7	Methyl petroselinate	C19H36O2	296	2166	21.947	85
F3	8	Methyl stearate	C19H38O2	298	2194	22.187	92
F10.C	9	1b,5,5,6a-Tetramethyl-octahydro-1-oxa-cyclopropa[a]inden-6-one	C13H20O2	208	2253	22.718	82
F10.C	10	4-Oxo-β-isodamascol	C13H20O2	208	2290	23.030	80
F10.C	11	Oleamide	C18H35NO	281	3188	29.548	92
F10.C	12	1,2-Octadecanediol	C18H38O2	286	3556	31.742	87
F10.C	13	Cyclopentadecanol	C15H30O	226	3796	33.665	94
F10.D	14	Dicyclohexane	C12H22	166	1269	11.733	94
F10.D	1	2,4-Di-tert-butylphenol	C14H22O	206	1450	14.300	89
F10.D	15	3-Dodecyl-2,5-furandione	C16H26O3	266	1825	18.630	86
F10.D	16	Methyl elaidate	C19H36O2	296	2031	20.680	94
F10.E	1	2,4-Di-tert-butylphenol	C14H22O	206	1450	14.297	91
F10.E	2	1-Octadecene	C18H36	252	1730	17.610	95
F10.E	17	n-Dodecenylsuccinic anhydride	C16H26O3	266	1832	18.697	86
F10.E	18	9-Eicosene	C20H40	280	2016	20.543	89
F10.E	19	11,14-Eicosadienoic acid, methyl ester	C21H38O2	322	2025	20.623	88
F10.E	20	Methyl oleate	C19H36O2	296	2031	20.683	93
Precipitate	21	Ascaridole	C10H16O2	168	2177	22.042	82
Precipitate	9	1b,5,5,6a-Tetramethyl-octahydro-1-oxa-cyclopropa[a]inden-6-one	C13H20O2	208	2253	22.718	82
Precipitate	22	1-Eicosanol	C20H42O	298	3557	31.750	90

After identification by GC–MS and comparison with NIST Mass Spectral Library, the chemical names are confirmed using PubChem [52].

**Table 4:** Biological comparison data of *C. papendorfii* crude extract and pure kheiric acid.

Strains	Crude Extract of *C. papendorfii*	Kheiric Acid
*Staphylococcus aureus*	MIC = 312 µg/mL	MIC = 62.5 µg/mL
MRSA	MIC = 312 µg/mL	MIC = 62.5 µg/mL
HCoV 229E	15% of the reduction of the virus-induced cytopathogenic effect at MOI 0.001	No reduction of the virus-induced cytopathogenic effect
FCV F9	40% of the reduction of the virus-induced cytopathogenic effect at lower MOI 0.0001	No reduction of the virus-induced cytopathogenic effect
MCF7	IC_50_ = 21.5 ± 5.9 µg/mL	IC_50_ > 100 µg/mL
HT29	IC_50_ > 100 µg/mL	IC_50_ > 100 µg/mL
HCT116	IC_50_ > 100 µg/mL	IC_50_ > 100 µg/mL

## Supplementary Materials

The following are available online at https://www.mdpi.com/2076-2607/8/9/1353/s1, Figure S1: Response-dose curves established from results obtained by MTT assays after MCF7, HT29 and HCT116 cells exposure of *C. papendorfii*; Figure S2: Chemical structure of compound **1**; Figure S3: IR spectrum of compound **1**; Figure S4: HR-ESI-MS spectrum of compound **1**; Figure S5: ^1^H-NMR spectrum of compound **1** in CD_3_OD (400 M Hz); Figure S6: ^13^C-NMR spectrum of compound **1** in CD_3_OD (100 M Hz); Figure S7: ^1^H-^1^H COSY spectrum of compound **1** in CD_3_OD; Figure S8: HSQC spectrum of compound **1** in CD_3_OD; Figure S9: HMBC spectrum of compound **1** in CD_3_OD; Figure S10: Zoom of HMBC spectrum of compound **1** in CD_3_OD; Figure S11: Zoom of HMBC spectrum of compound **1** in CD_3_OD; Figure S12: ^1^H-NMR spectrum of compound **1** in pyridine *d_5_* (400 M Hz); Figure S13: ^13^C-NMR spectrum of compound **1** in pyridine *d_5_* (100 M Hz); Figure S14: Chemical structure of compound **2**; Figure S15: HR-ESI-MS spectrum of compound **2**; Figure S16: ^1^H-NMR spectrum of compound **2** in DMSO *d_6_* (400 M Hz); Figure S17: ^13^C-NMR spectrum of compound **2** in DMSO *d_6_* (100 M Hz); Figure S18: ^1^H-^1^H COSY spectrum of compound **2**; Figure S19: HSQC spectrum of compound **2**; Figure S20: HMBC spectrum of compound **2**.
