# Supplementary material for: Evaluation of Antiviral, Antibacterial and Antiproliferative Activities of the Endophytic Fungus Curvularia papendorfii, and Isolation of a New Polyhydroxyacid
Source: Microorganisms. 2020 Sep 4;8(9):1353. doi: 10.3390/microorganisms8091353 (PMC7564059; doi:10.3390/microorganisms8091353)

# Supplementary materials

## Evaluation of antiviral, antibacterial and antiproliferative activities of the endophytic fungus *Curvularia papendorfii*, and isolation of a new polyhydroxyacid.

**Afra Khiralla[1,2], Rosella Spina[1,*] , Mihayl Varbanov[1], Stéphanie Philippot[1], Pascal Lemiere[1], Sophie Slezack-Deschaumes[3], Philippe André[4], Ietidal Mohamed[5], Sakina Mohamed Yagi[5] and Dominique Laurain-Mattar [1,*]**

[1] Université de Lorraine, CNRS, L2CM, F-54000 Nancy, France; aafraa21@hotmail.com (A.K), rosella.spina@univ-lorraine.fr (R.S), mihayl.varbanov@univ-lorraine.fr (M.V.), stephanie.philippot@univ-lorraine.fr (S.P.), pascal.lemiere@univ-lorraine.fr (P.L), dominique.mattar@univ-lorraine.fr (D.L.-M.)
[2] Botany Department, Faculty of Sciences and technologies, Shendi University, P. O. Box 142, Shendi, Sudan.; aafraa21@hotmail.com (A.K)
[3] Université de Lorraine, Inra, LAE, F-54000 Nancy, France.; sophie.deschaumes@univ-lorraine.fr (S.S.-D.)
[4] Université de Strasbourg, UMR 7021 CNRS, 67401 Illkirch, France; philippe.andre@unistra.fr (P.A.)
[5] Department of Botany, Faculty of Science, University of Khartoum, 11115 Khartoum, Sudan; sakinayagi@gmail.com (S.Y.), ietidalem11@gmail.com (I.M.)

***** Correspondence: rosella.spina@univ-lorraine.fr (R.S. Tel.: +33-3-7274-5226 (Orcid 0000-0003-0969-2587) and dominique.mattar@univ-lorraine.fr (D.L.-M.) Tel.: +33-3-7274-5675 (Orcid 0000-0001-7013-8505)

# Contents

**Figure S1**: Response-dose curves established from results obtained by MTT assays after MCF7, HT29 and HCT116 cells exposure of *C. papendorfii*.
**Figure S2**: Chemical structure of compound **1**
**Figure S3**: IR spectrum of compound **1**
**Figure S4**: HR-ESI-MS spectrum of compound **1**
**Figure S5**: $^{1}$H-NMR spectrum of compound **1** in $CD_3OD$ (400 MHz)
**Figure S6**: $^{13}$C-NMR spectrum of compound **1** in $CD_3OD$ (100 MHz)
**Figure S7**: $^{1}$H-$^{1}$H COSY spectrum of compound **1** in $CD_3OD$
**Figure S8**: HSQC spectrum of compound **1** in $CD_3OD$
**Figure S9**: HMBC spectrum of compound **1** in $CD_3OD$
**Figure S10**: Zoom of HMBC spectrum of compound **1** in $CD_3OD$
**Figure S11**: Zoom of HMBC spectrum of compound **1** in $CD_3OD$
**Figure S12**: $^{1}$H-NMR spectrum of compound **1** in pyridine $d_5$ (400 MHz).
**Figure S13**: $^{13}$C-NMR spectrum of compound **1** in pyridine $d_5$ (100 MHz)
**Figure S14**: Chemical structure of compound **2**
**Figure S15**: HR-ESI-MS spectrum of compound **2**
**Figure S16**: $^{1}$H-NMR spectrum of compound **2** in DMSO $d_6$ (400 MHz).
**Figure S17**: $^{13}$C-NMR spectrum of compound **2** in DMSO $d_6$ (100 MHz)
**Figure S18**: $^{1}$H-$^{1}$H COSY spectrum of compound **2**
**Figure S19**: HSQC spectrum of compound **2**
**Figure S20**: HMBC spectrum of compound **2**

**Figure S1**: Response-dose curves established from results obtained by MTT assays after MCF7, HT29 and HCT116 cells exposure of *C. papendorfii*.

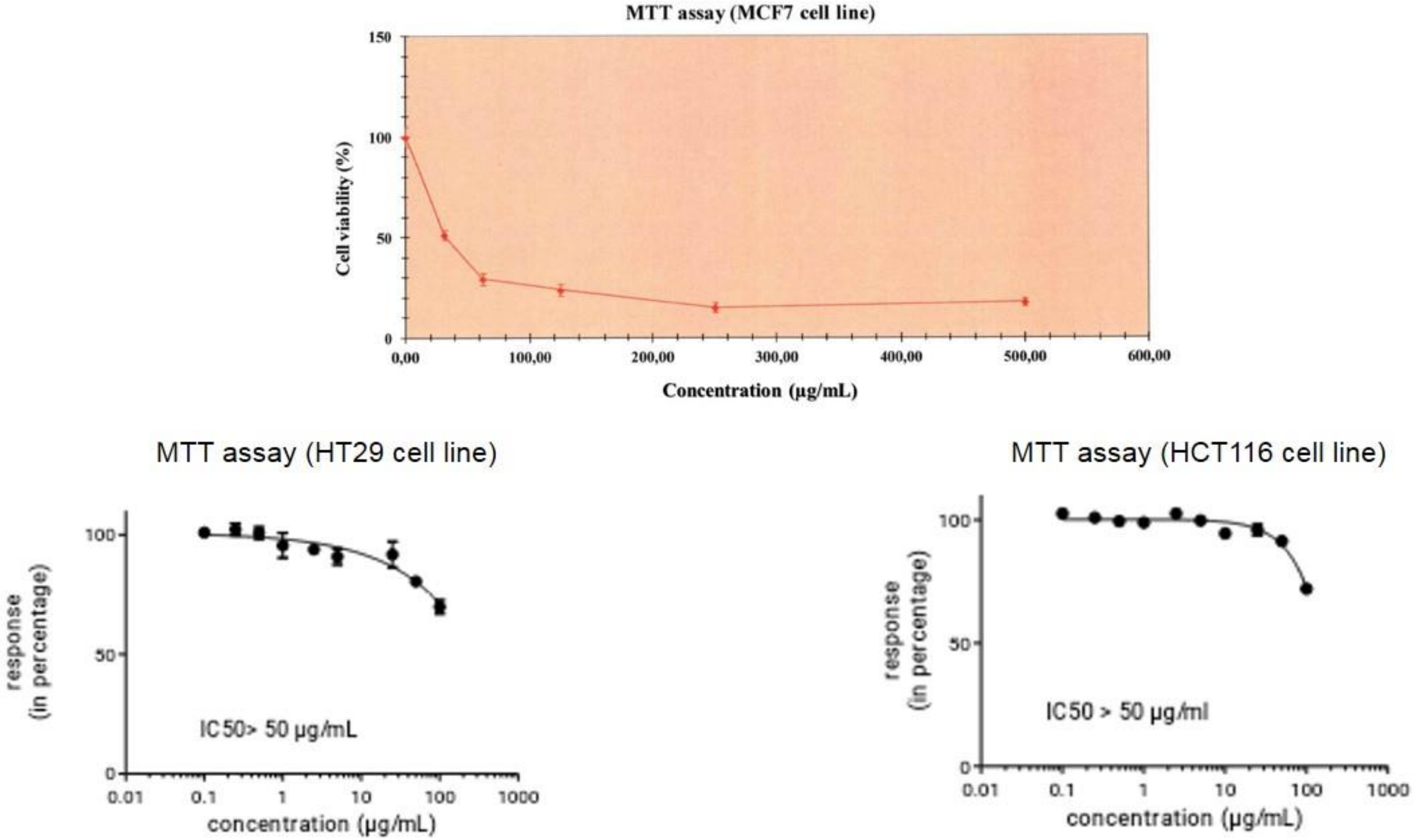

**Figure S2**: Chemical structure of compound **1**

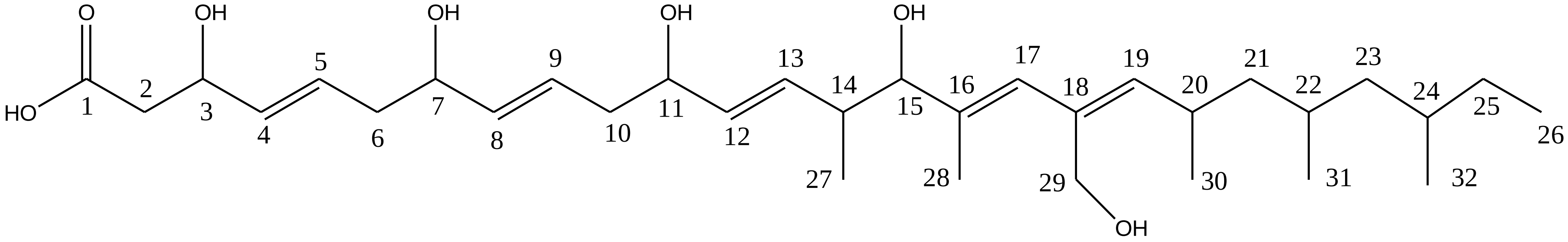


3,7,11,15-Tetrahydroxy-18-hydroxymethyl-14,16,20,22,24-pentamethyl-hexacosa-4E,8E,12E,16,18-pentaenoic acid

=

kheiric acid

**Figure S3:** IR spectrum of compound **1**

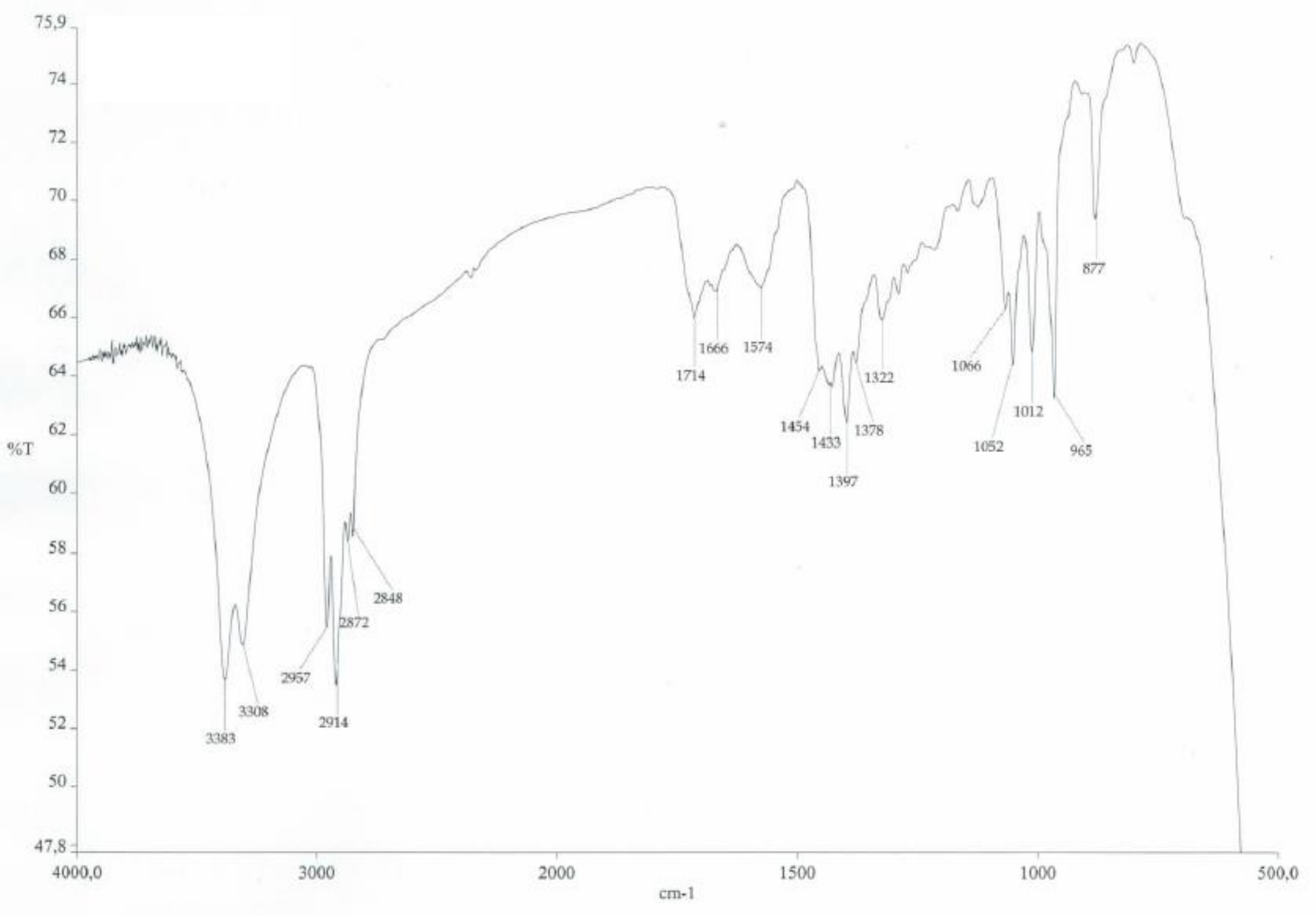

**Figure S4**: HR-ESI-MS spectrum of compound **1**

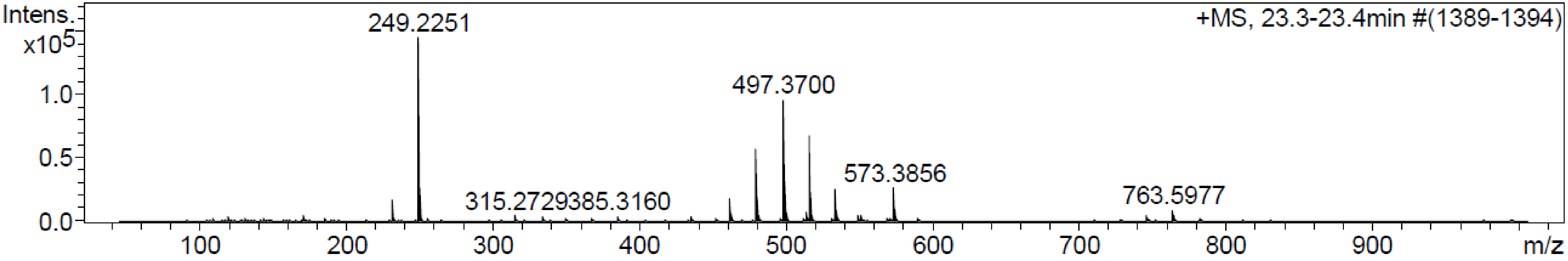

**Figure S5**: $^1$H-NMR spectrum of compound **1** in $CD_3OD$ (400 MHz)

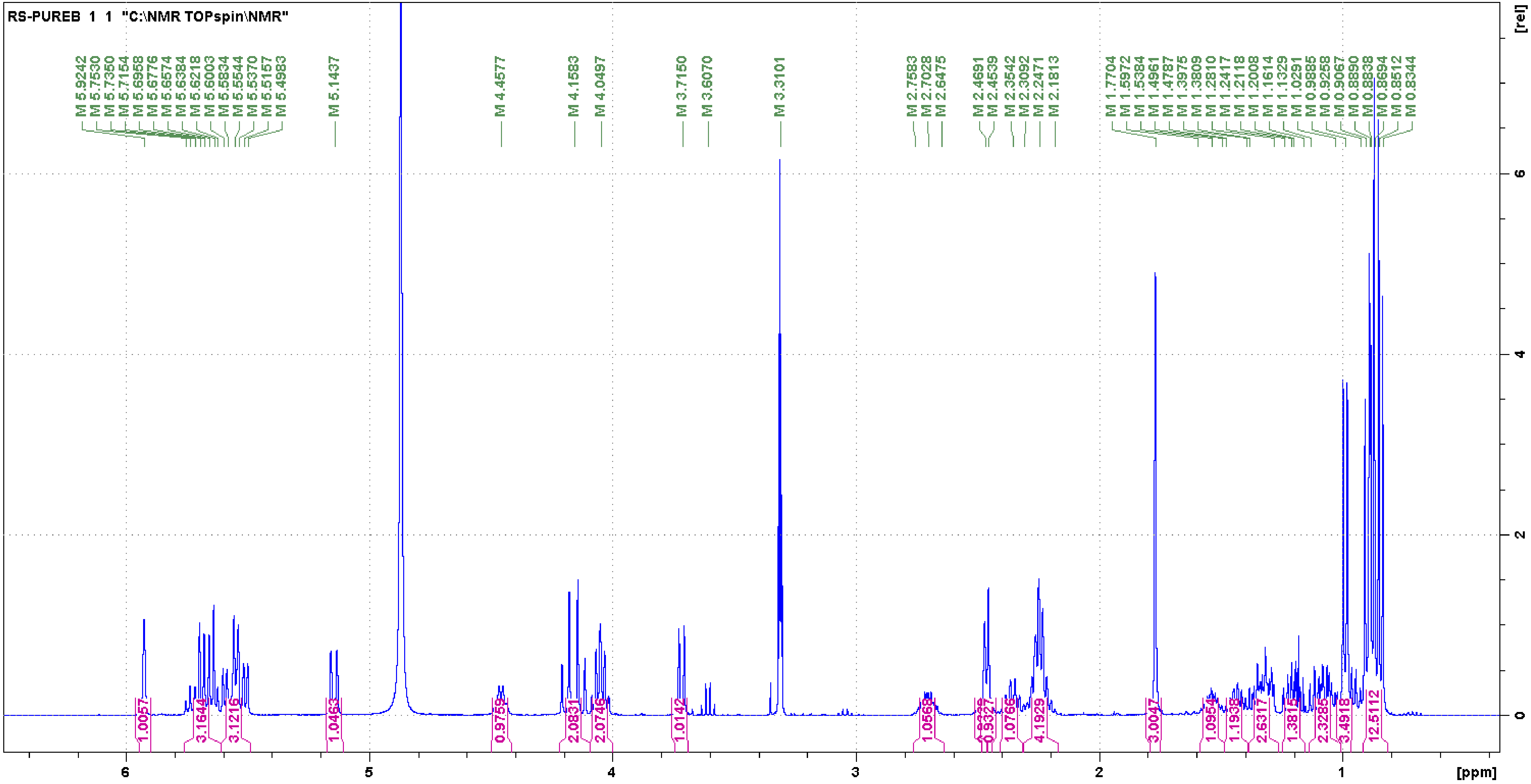

**Figure S6**: $^{13}C$-NMR spectrum of compound **1** in $CD_3OD$ (100 MHz)

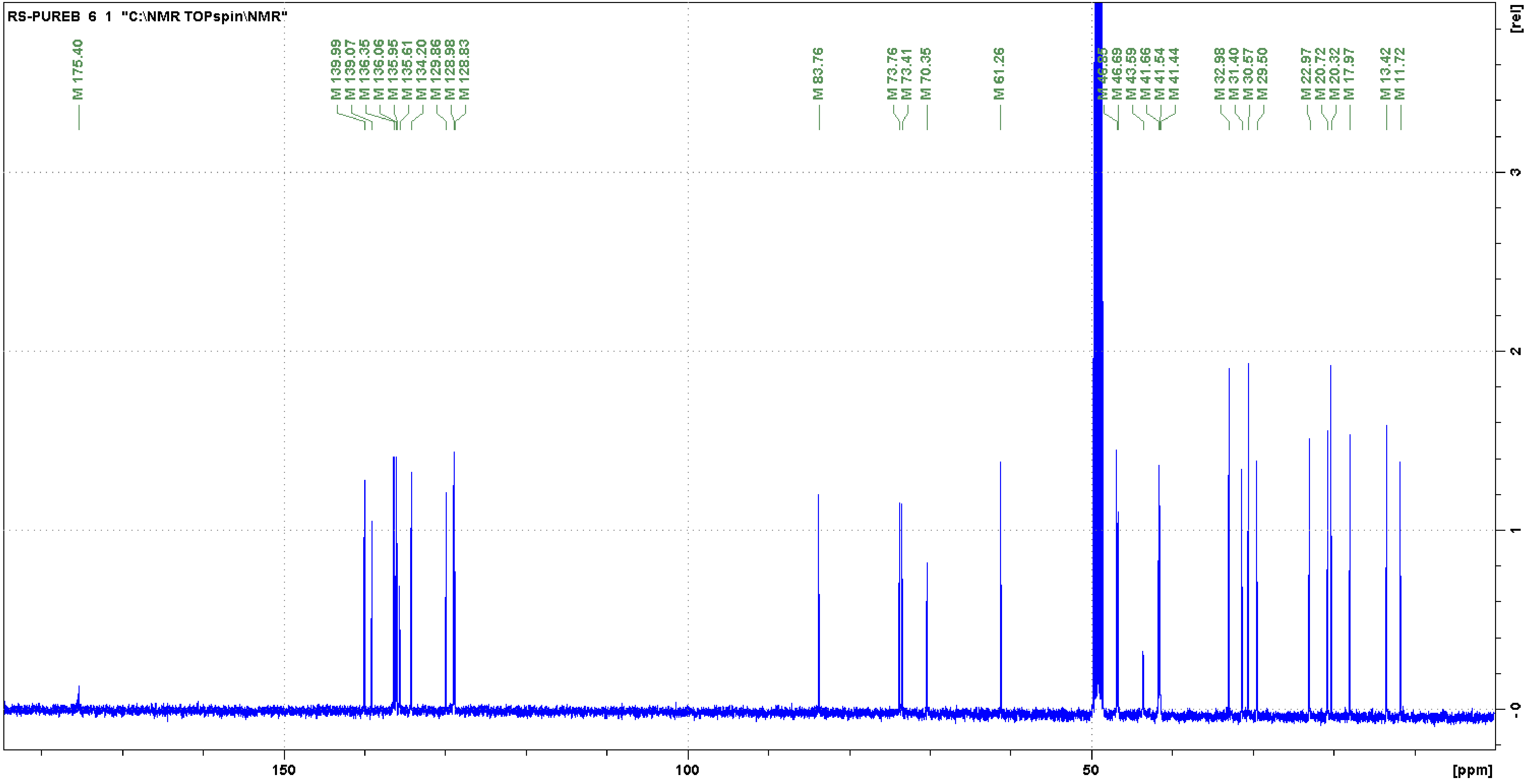

**Figure S7**: $^{1}H$-$^{1}H$ COSY spectrum of compound **1** in $CD_3OD$

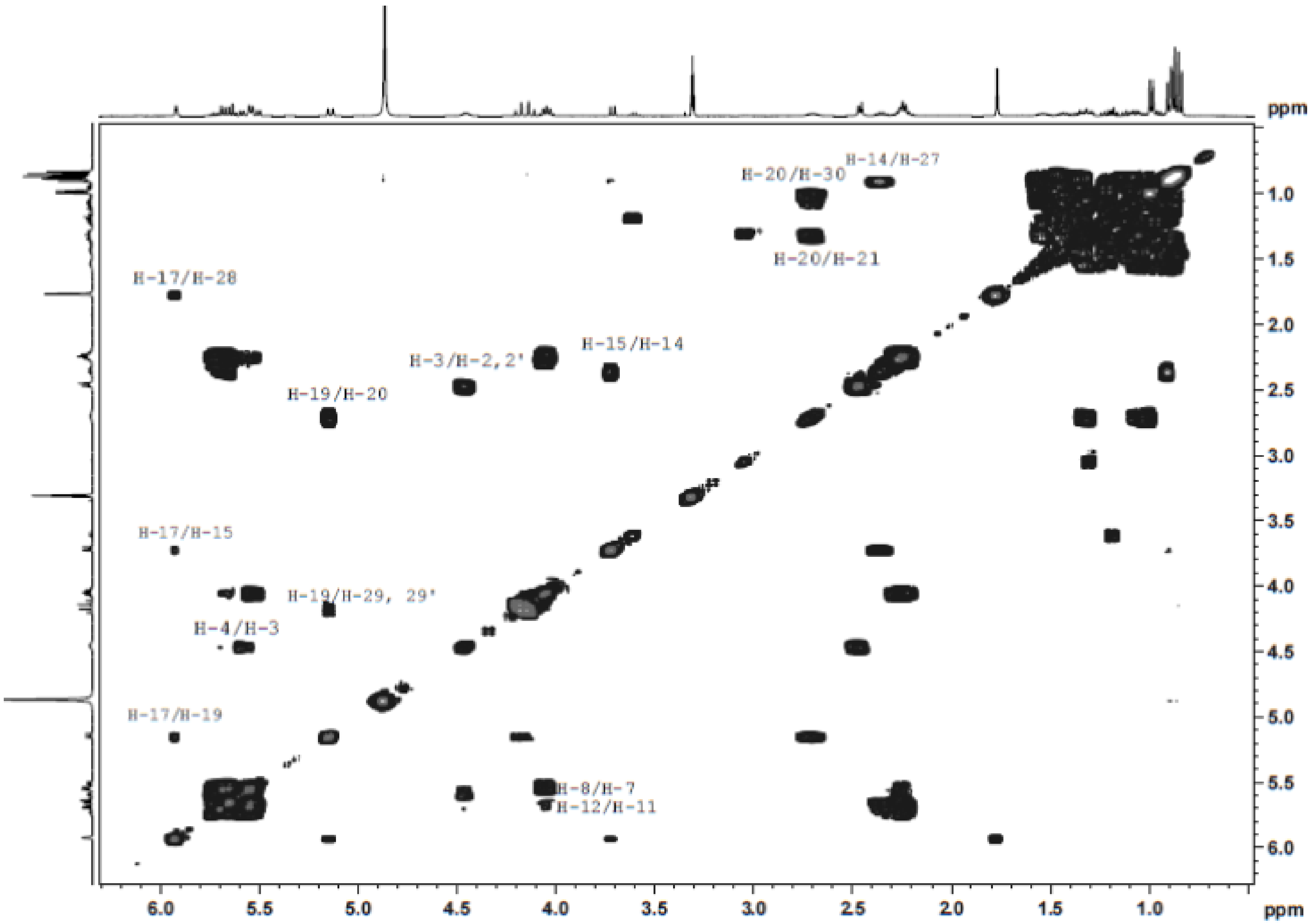

**Figure S8**: HSQC spectrum of compound **1** in $CD_3OD$

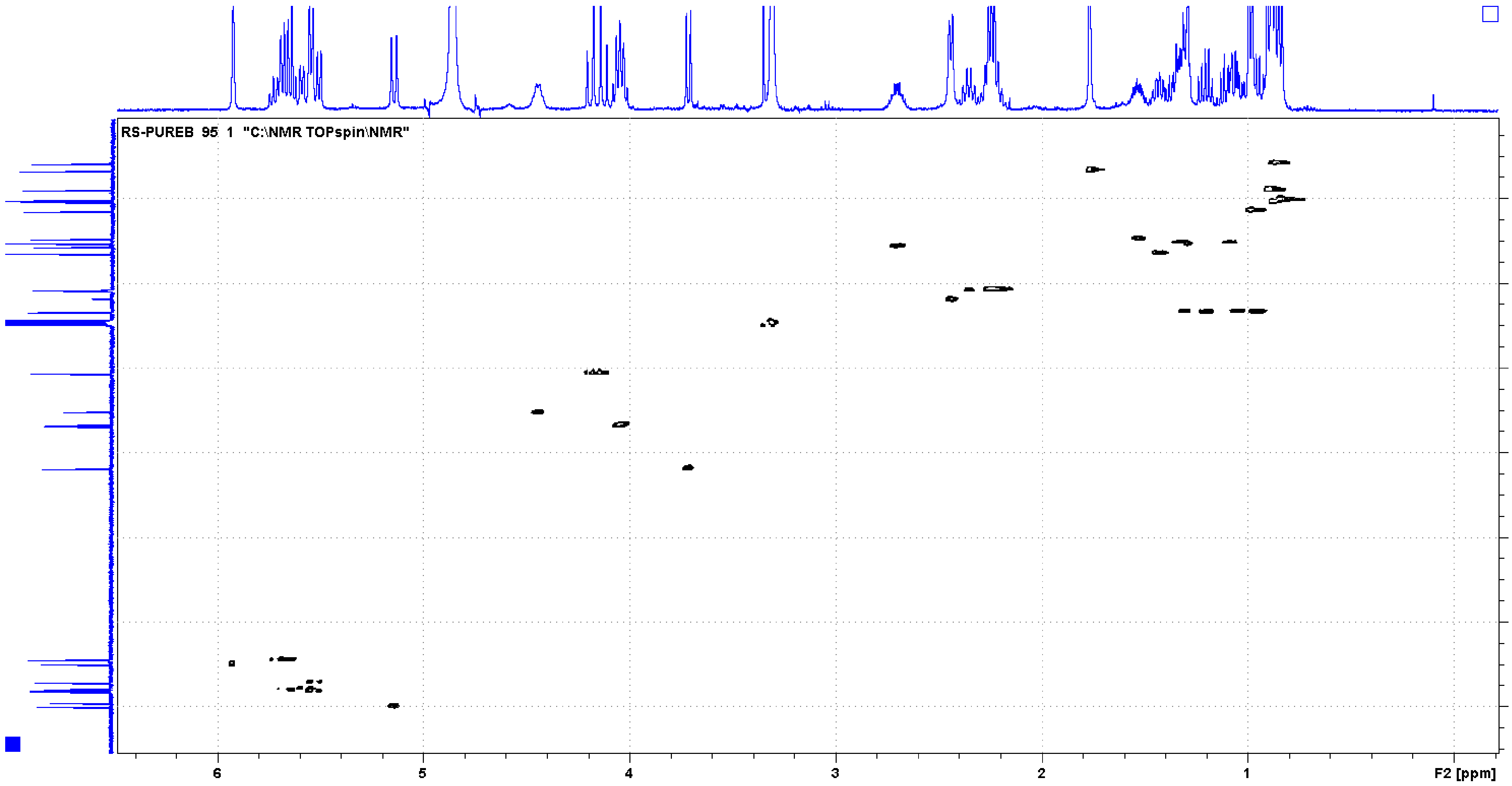

**Figure S9**: Zoom HSQC spectrum of compound **1** in $CD_3OD$

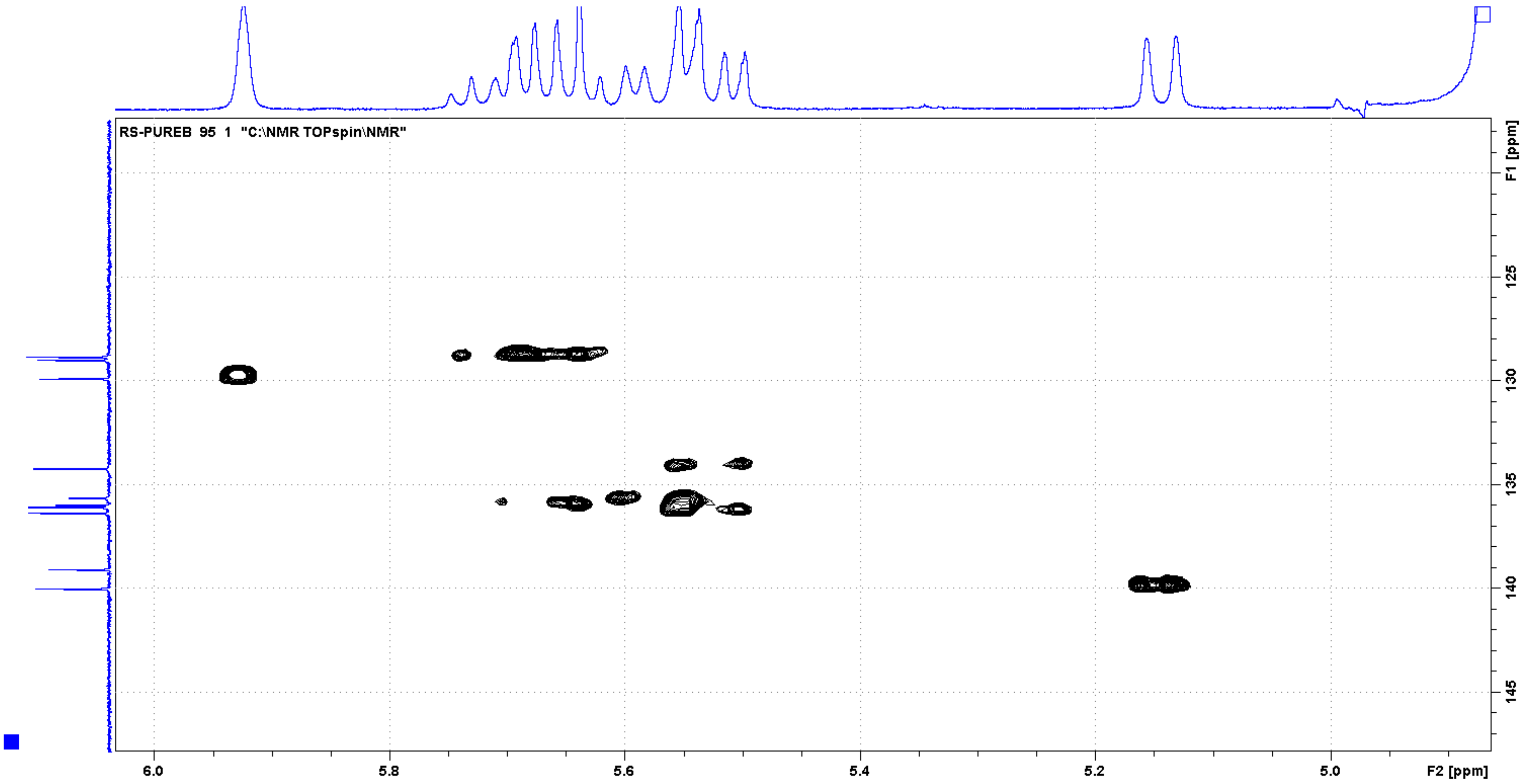

**Figure S10**: HMBC spectrum of compound **1** in $CD_3OD$

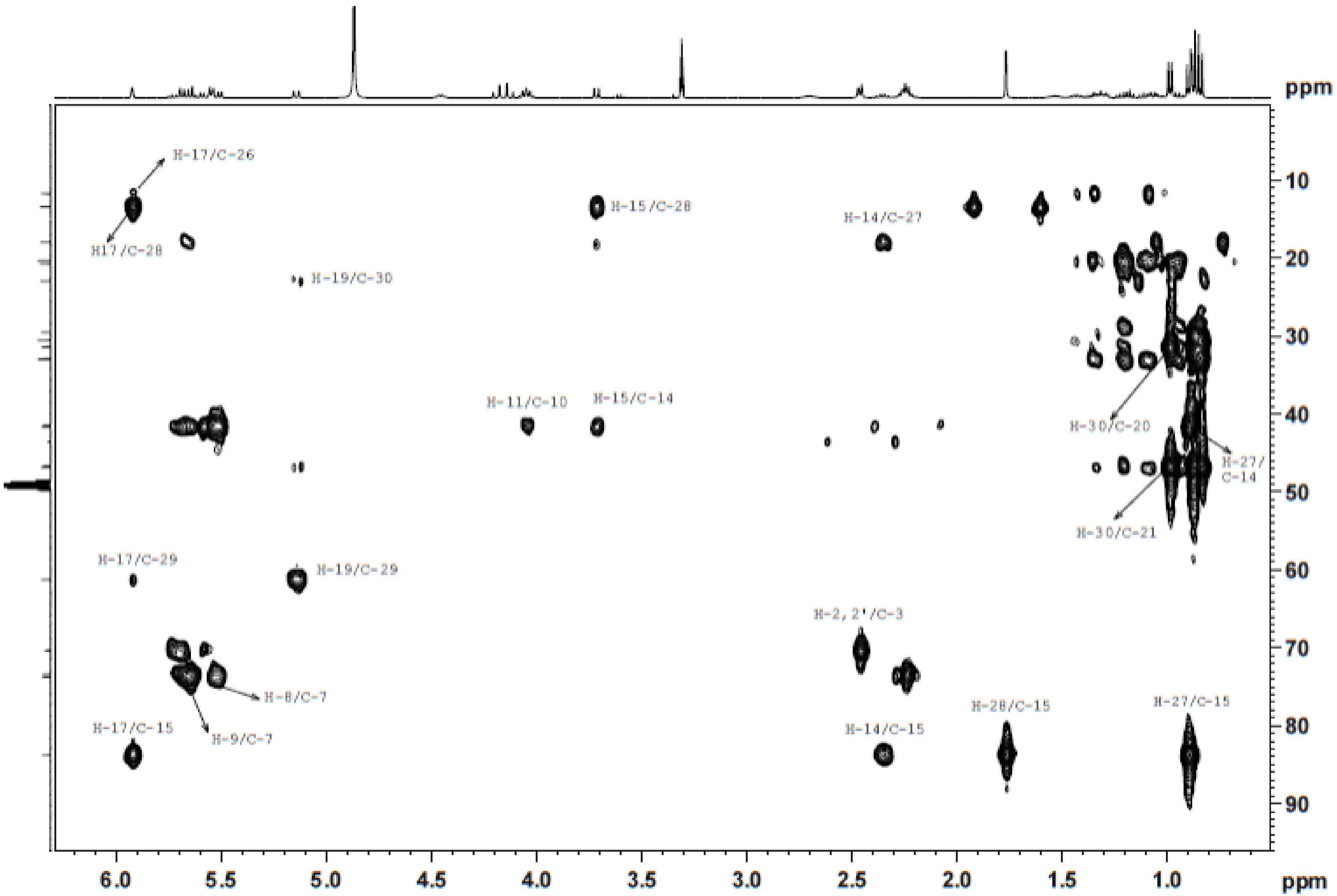

**Figure S11**: Zoom of HMBC spectrum of compound **1** in $CD_3OD$

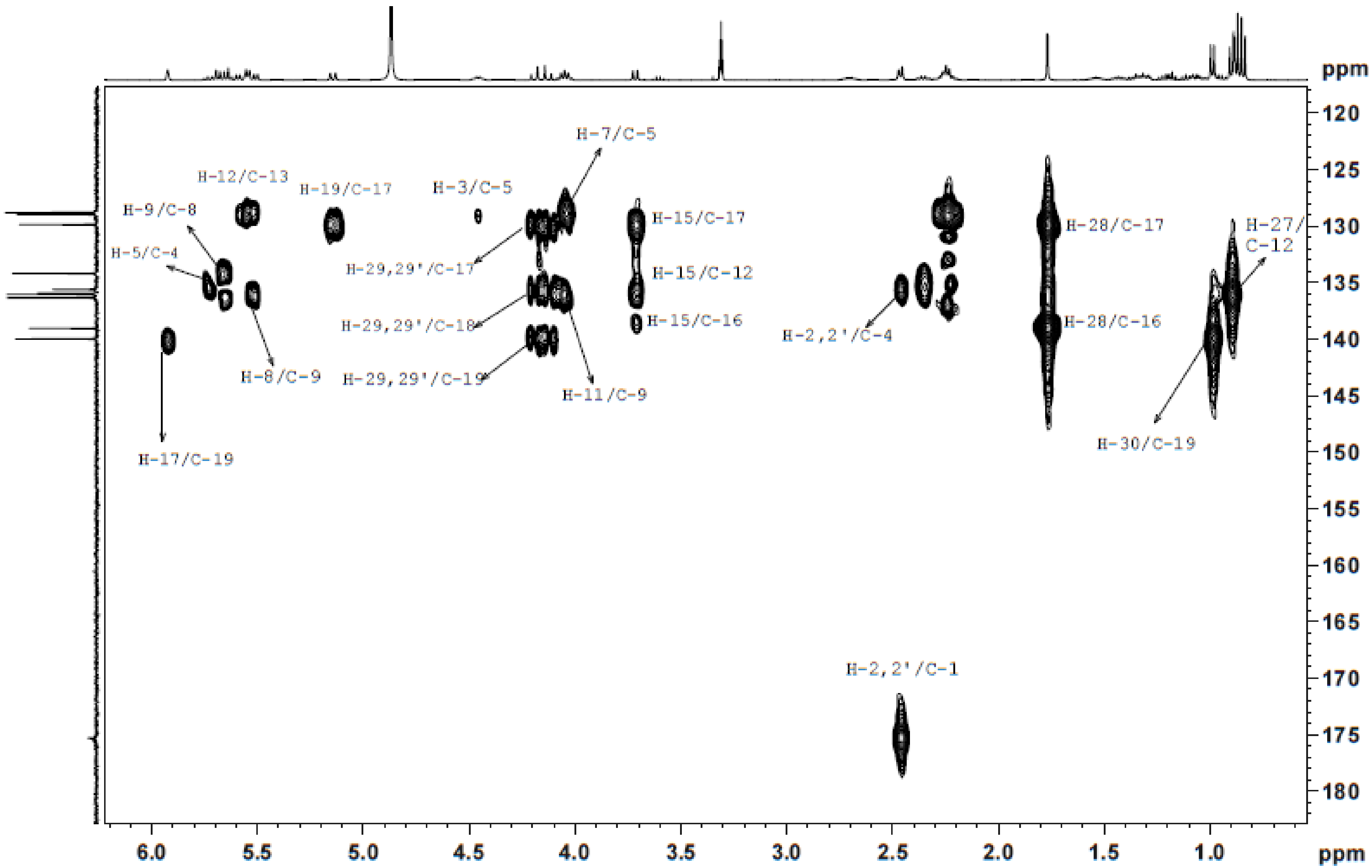

**Figure S12**: $^{1}$H-NMR spectrum of compound **1** in pyridine $d_5$ (400 MHz)

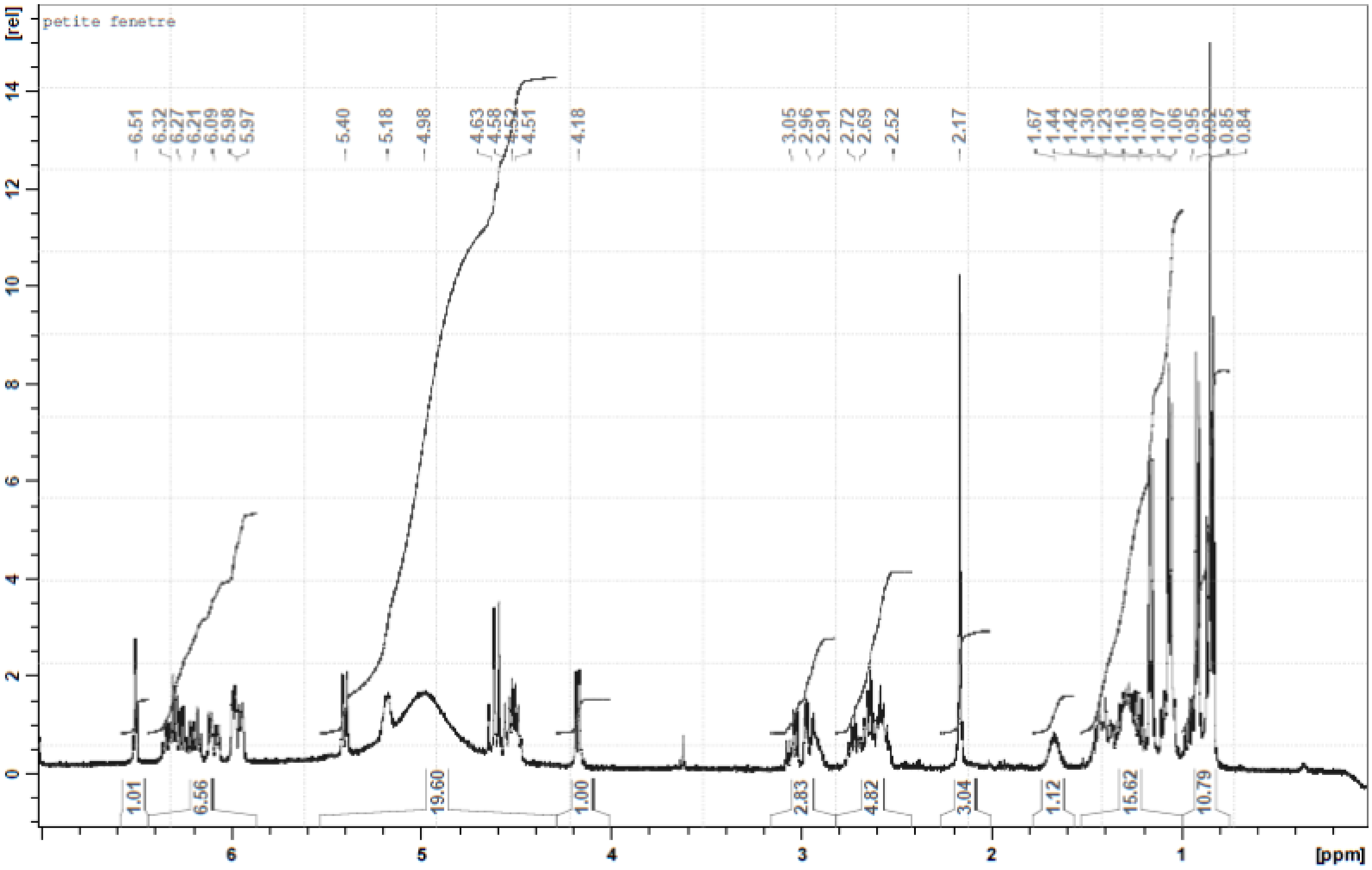

**Figure S13**: $^{13}$C-NMR spectrum of compound **1** in pyridine $d_5$ (100 MHz)

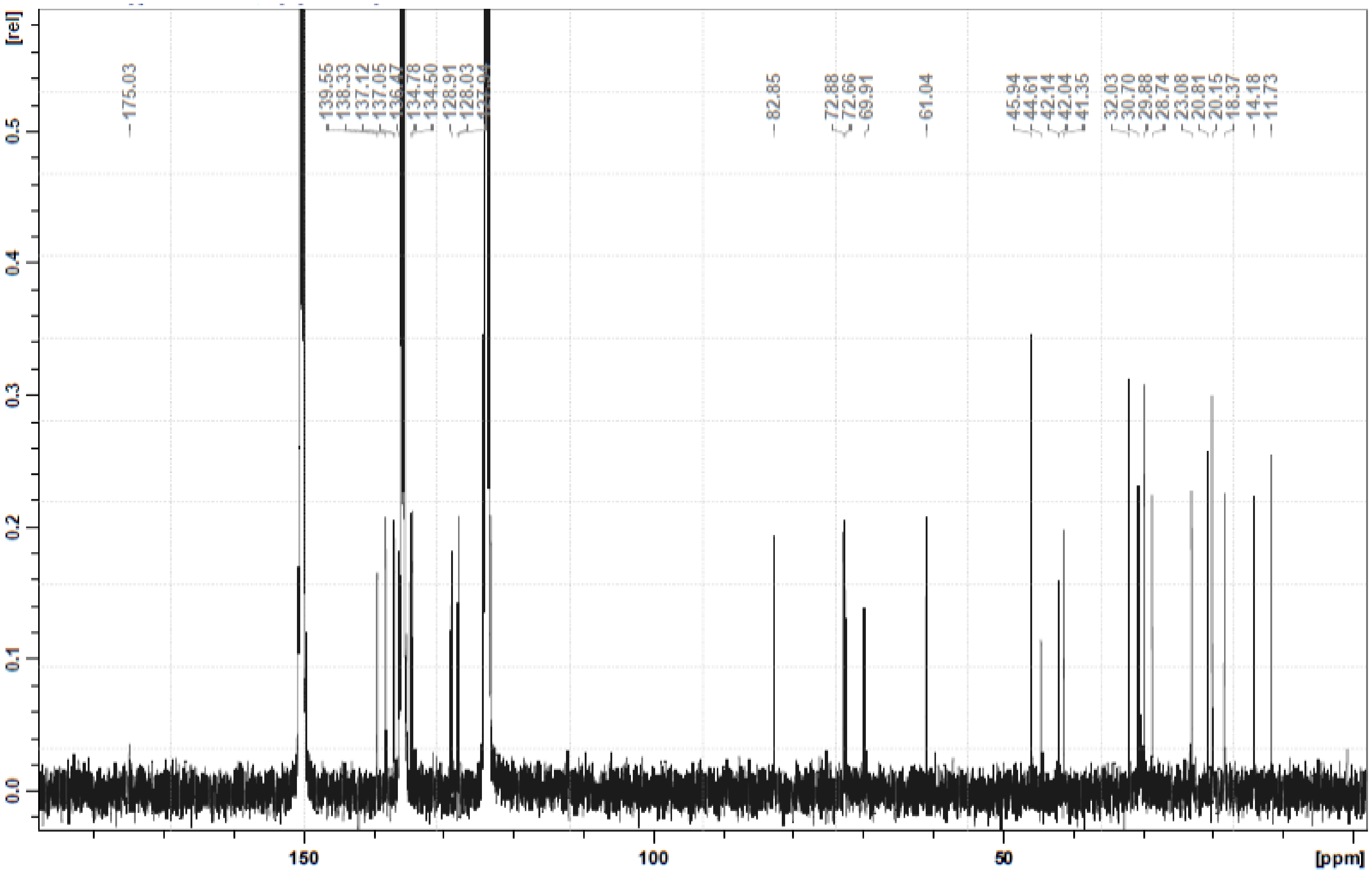

**Figure S14:** Chemical structure of compound **2**

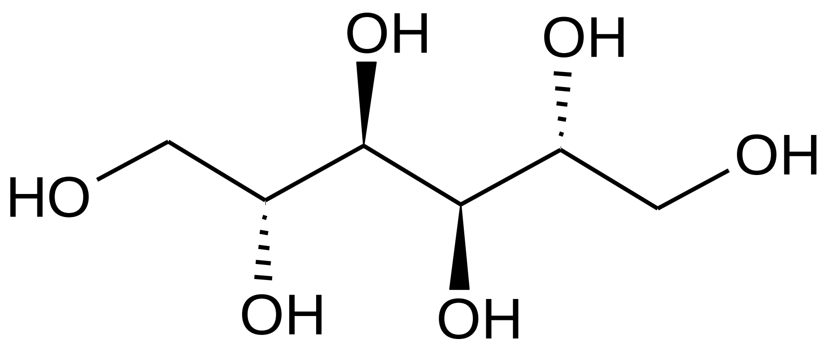


Mannitol

**Figure S15**: HR-ESI-MS spectrum of compound **2**

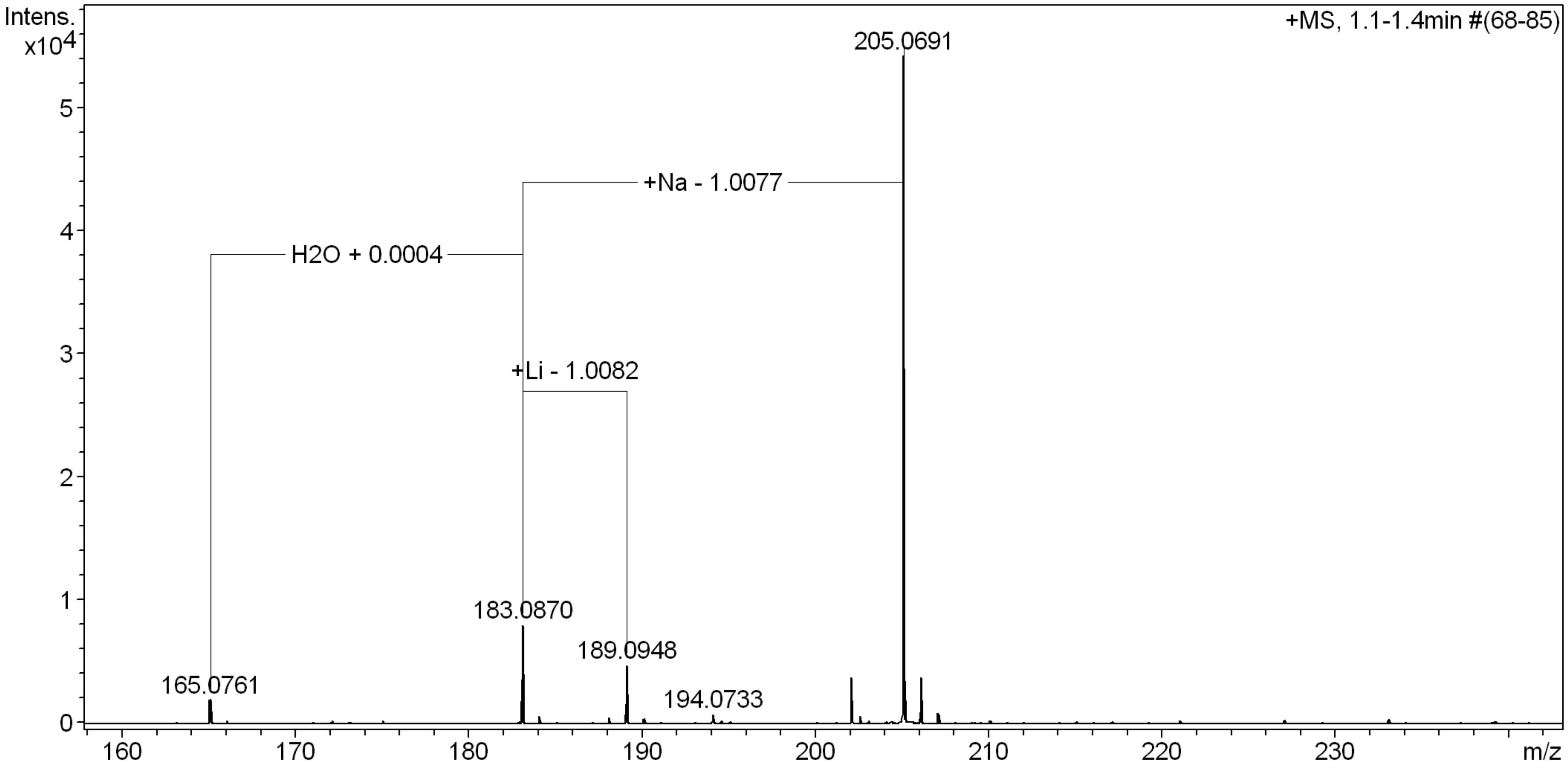

**Figure S16**: $^1$H-NMR spectrum of compound **2** in DMSO $d_6$ (400 MHz)

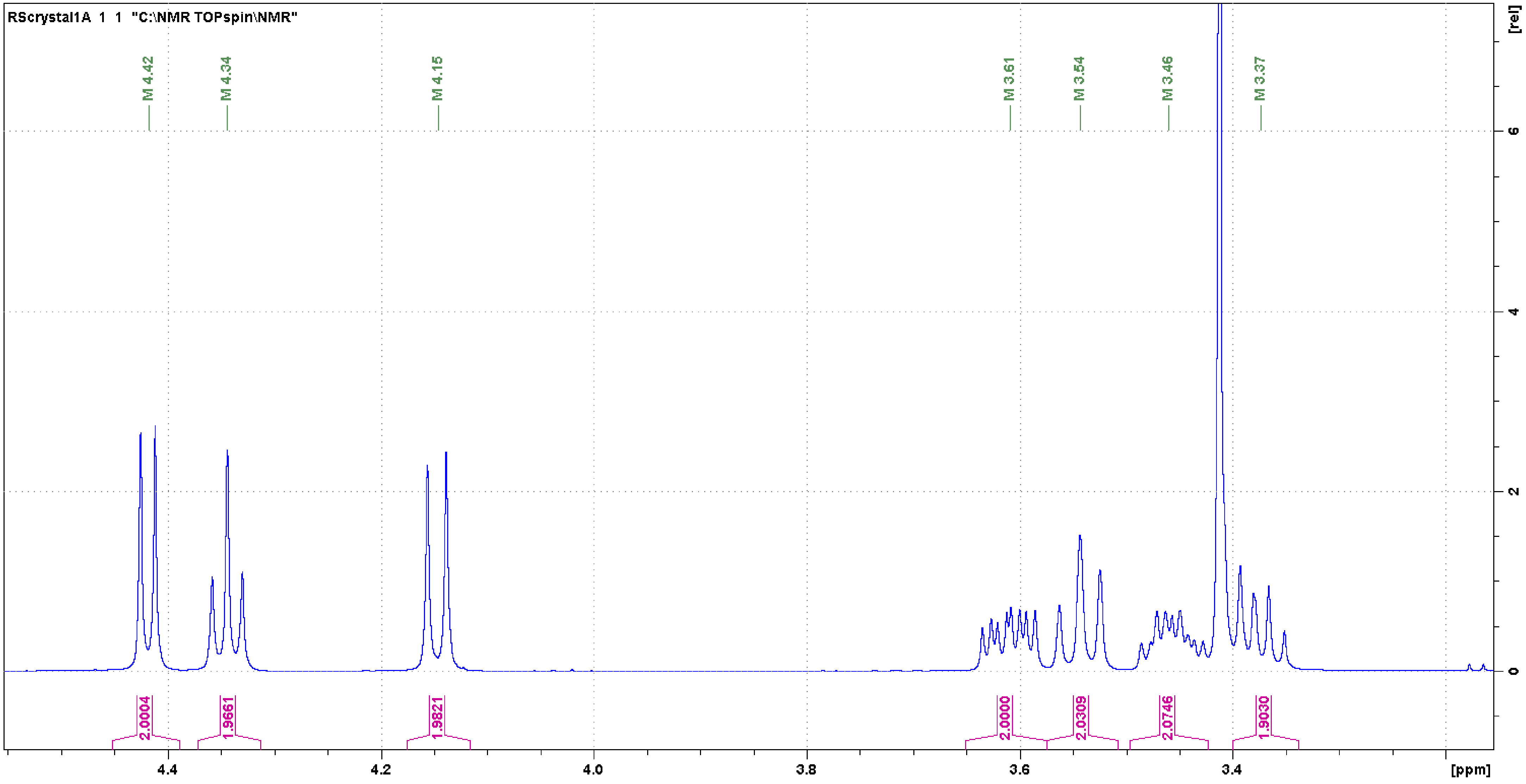

**Figure S17**: $^{13}$C-NMR spectrum of compound **2** in DMSO $d_6$ (100 MHz)

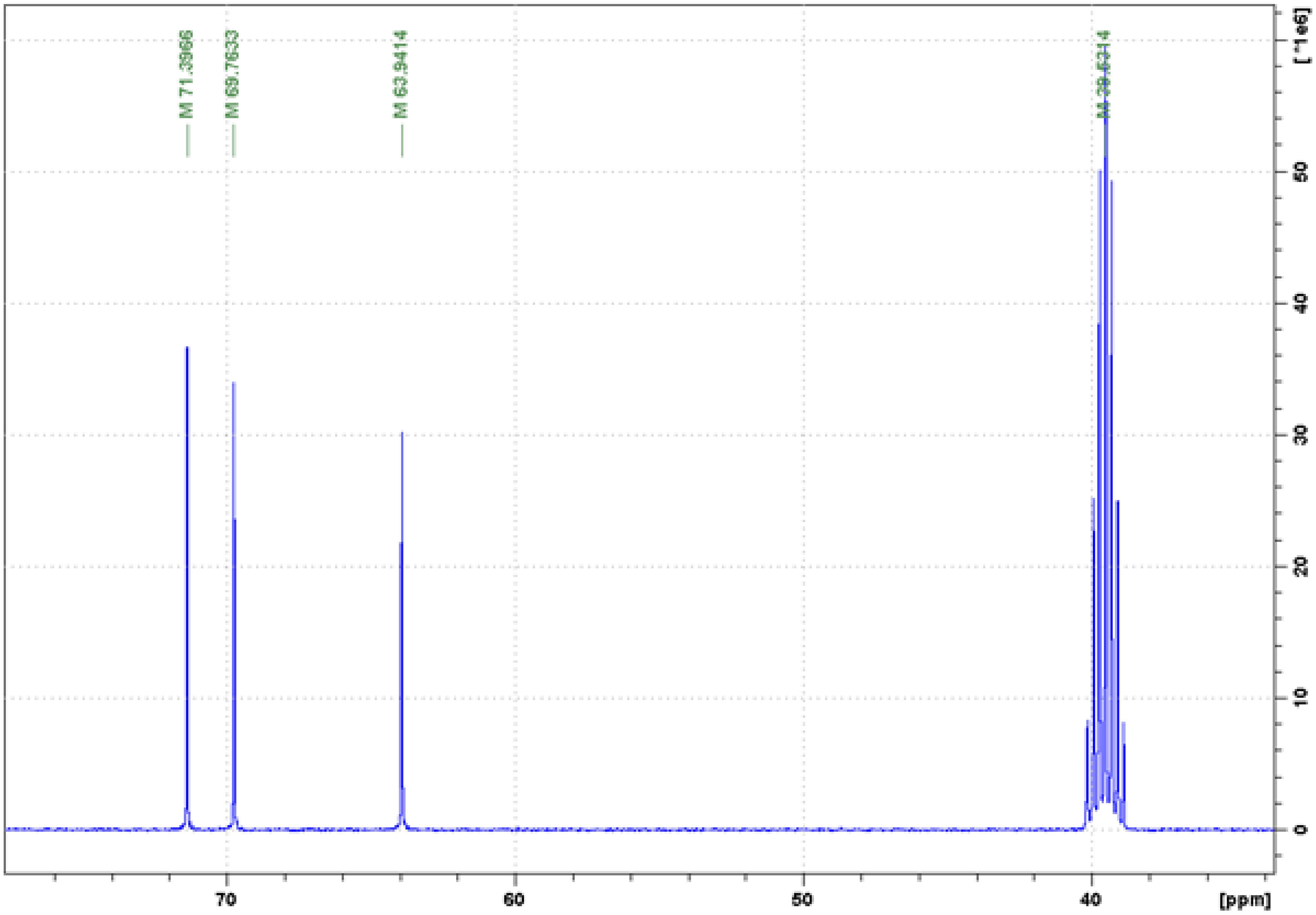

**Figure S18**: $^{1}H$-$^{1}H$ COSY spectrum of compound **2**

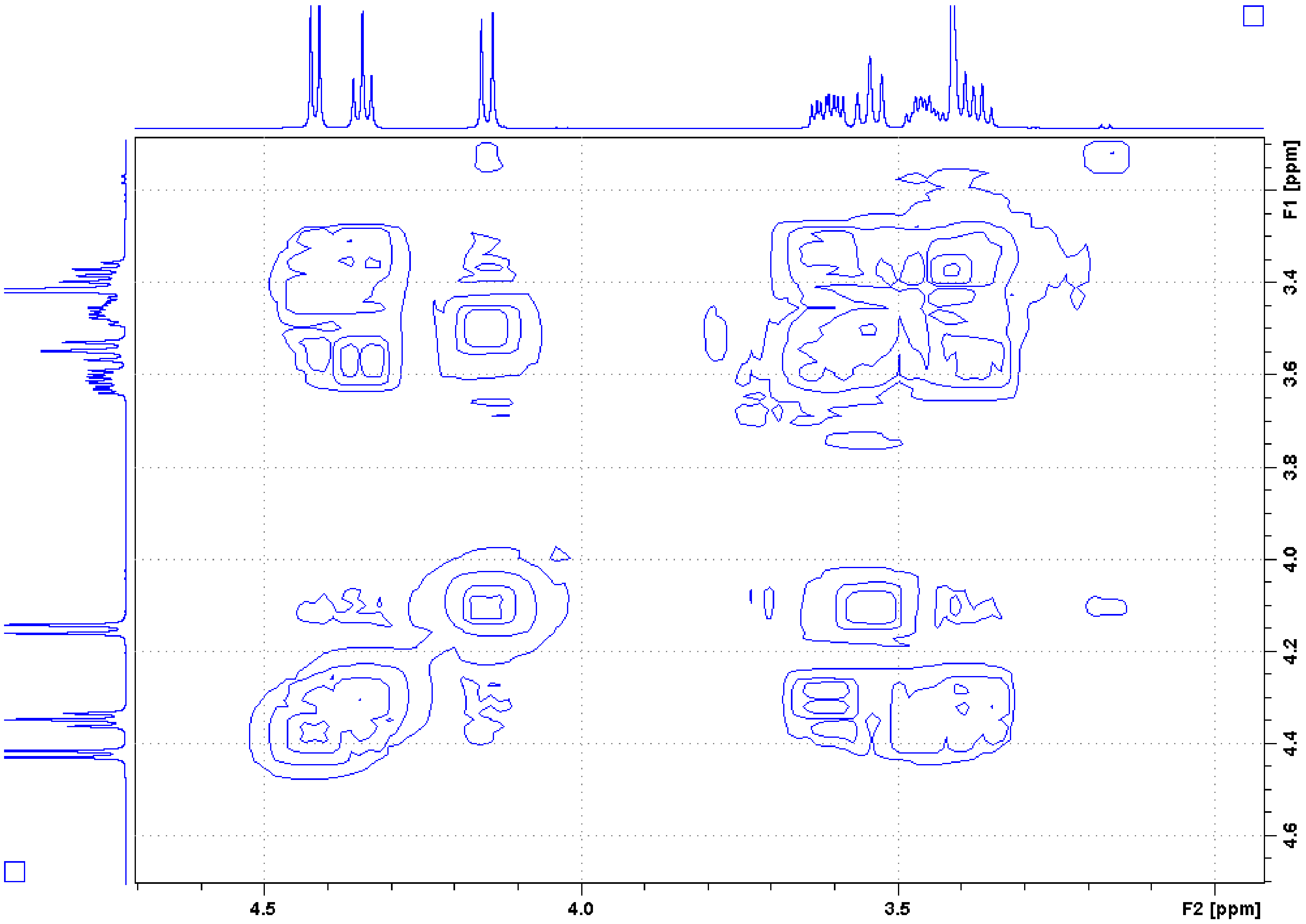

**Figure S19**: HSQC spectrum of compound **2**

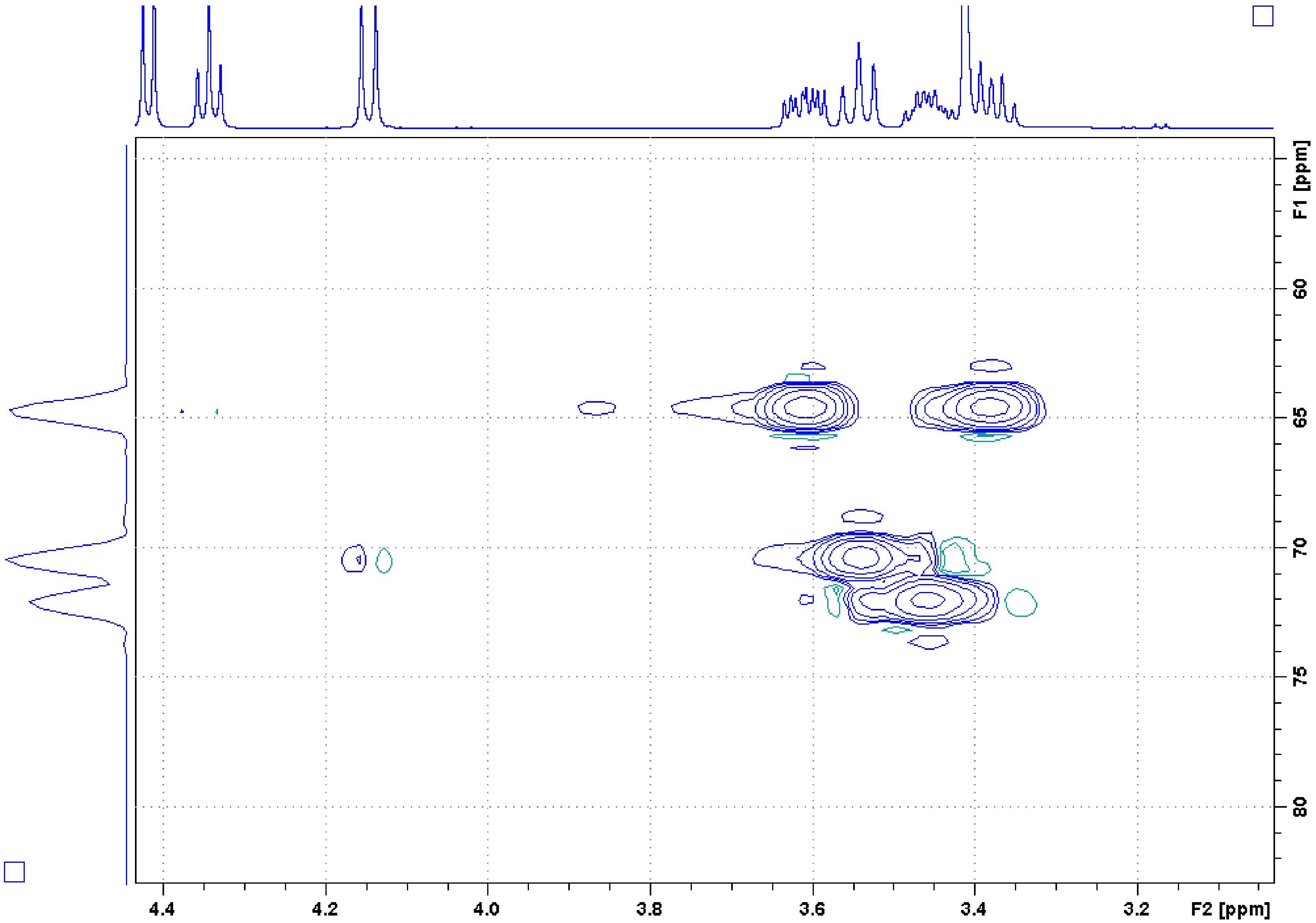

**Figure S20**: HMBC spectrum of compound **2**

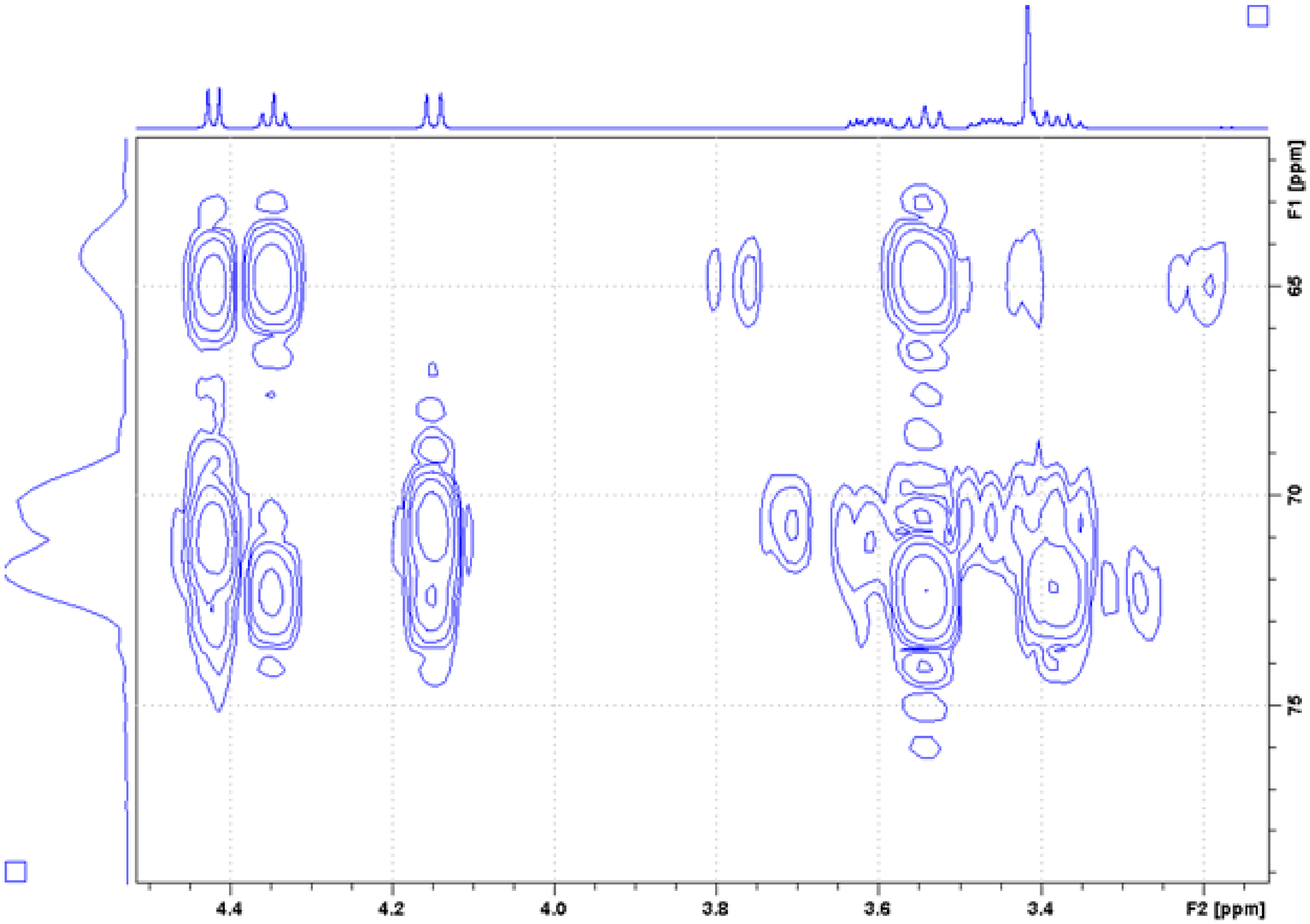

Supplement: Supplementary file 1 [file microorganisms-08-01353-s001.pdf]
